# Spatio-temporal expression pattern and role of the tight junction protein MarvelD3 in pancreas development and function

**DOI:** 10.1038/s41598-021-93654-2

**Published:** 2021-07-15

**Authors:** Charlotte Heymans, Ophélie Delcorte, Catherine Spourquet, Mylah Villacorte-Tabelin, Sébastien Dupasquier, Younes Achouri, Siam Mahibullah, Pascale Lemoine, Maria S. Balda, Karl Matter, Christophe E. Pierreux

**Affiliations:** 1 Cell Biology Unit, de Duve Institute, UCLouvain, Woluwe, Belgium; 2 PRISM, MSU-IIT, Iligan City, Philippines; 3 Transgenesis Platform, UCLouvain, Woluwe, Belgium; 4 Institute of Ophthalmology, UCL, London, UK

**Keywords:** Tight junctions, Differentiation, Organogenesis

## Abstract

Tight junction complexes are involved in the establishment and maintenance of cell polarity and the regulation of signalling pathways, controlling biological processes such as cell differentiation and cell proliferation. MarvelD3 is a tight junction protein expressed in adult epithelial and endothelial cells. In *Xenopus laevis,* MarvelD3 morphants present differentiation defects of several ectodermal derivatives. In vitro experiments further revealed that MarvelD3 couples tight junctions to the MEKK1-JNK pathway to regulate cell behaviour and survival. In this work, we found that MarvelD3 is expressed from early developmental stages in the exocrine and endocrine compartments of the pancreas, as well as in endothelial cells of this organ. We thoroughly characterized MarvelD3 expression pattern in developing pancreas and evaluated its function by genetic ablation. Surprisingly, inactivation of MarvelD3 in mice did not alter development and differentiation of the pancreatic tissue. Moreover, tight junction formation and organization, cell polarization, and activity of the JNK-pathway were not impacted by the deletion of MarvelD3.

## Introduction

Tight junctions (TJs) are multiprotein complexes composed of integral membrane proteins connected to underlying cytoplasmic plaque proteins (such as Zonula Occludens proteins), and are located at the apico-lateral border of neighbouring epithelial cells^1^. The transmembrane proteins ensure the interaction of TJ strands between adjacent cells, thus sealing the paracellular space and regulating the permeability of the epithelial sheet to ions and macromolecules^2,3^. TJs also contribute to the establishment and the maintenance of epithelial polarity by forming a fence that prevents diffusion of lipids and proteins within the plasma membrane^4,5^. Finally, TJs regulate signalling pathways involved in cell proliferation, survival or differentiation, through interactions with plaque proteins^4,6^.

The Tight junction Associated Marvel Proteins (TAMP) are transmembrane proteins that share a common structural domain: the Marvel domain, or *MAL and related proteins for vesicle trafficking and membrane link* domain, which is composed of four transmembrane α-helices that span the lipid bilayer in an M-shape^7^. Proteins containing this domain can be classified into four families: the MAL proteins, the gyrins, the physins and the occludin or TAMP proteins^8^. While the exact role of the Marvel domain has not yet been clearly established, it has been demonstrated that several of the Marvel-containing proteins play a role in cholesterol-rich membrane fusion or apposition events^9^, while others participate in tight junction function^10–13^. The TAMP family consists of occludin, tricellulin (also called MarvelD2) and MarvelD3^14^. The latter is a ± 43 kDa protein existing in two splice variants, MD3.1 and MD3.2^14,15^, which are expressed in a variety of epithelial and endothelial cell lines and in several adult mouse tissues, with apparent differences in their relative abundance^14,15^. Knock-down experiments in intestinal Caco-2 cells have shown that MarvelD3 is not implicated in the formation of TJs, but rather plays a role in TJ permeability and stabilization^14,15^. Moreover, in vivo depletion of MarvelD3 using antisense morpholinos in *Xenopus laevis* embryos results in alteration of the development of ectoderm-derived tissues such as the eye or the cranio-facial cartilage, and reduction in lateral line pigmentation of the skin. This is explained by molecular interactions of MarvelD3 with the MAP kinase kinase MEKK1, which fine-tunes the JNK-pathway in order to regulate development^16,17^. Finally, it has been reported that MarvelD3 intracellularly interacts with occludin and tricellulin and other tight junction proteins of the claudin family, namely claudin-1, 3, -4 and -5^18^.

The exocrine pancreas is an epithelial tissue composed of acinar and ductal cells, which produce and transport digestive enzymes, respectively. The pancreatic epithelium consists of a monolayer of polarized cells organized in a tree-like branched structure where acinar cells assemble into ovoid acini opening up into pancreatic ducts^19^. During the early stages of development, proliferation of pancreatic progenitors first generates a mass of non-polarized cells^20,21^. With time, cells express junctional proteins, which are necessary to initiate epithelial polarization^22^, and organize into a branched and polarized monolayer. Later, maintenance of cell polarity assures proper development, differentiation and function of the exocrine tissue^23^.

In the present study, we determined the spatio-temporal expression pattern of MarvelD3 in the developing mouse embryo, and generated a total knockout using the CRISPR-Cas9 technique to elucidate the role of MarvelD3. MarvelD3 was highly expressed in the pancreas, and salivary glands, as compared to other developing epithelia. However, pancreas histology, differentiation and function were normal in the absence of MarvelD3. Moreover, the abundance and localisation of several tight junction components were unaffected in the absence of MarvelD3. Finally, the activity of the JNK-pathway in the pancreas was not altered upon MarvelD3 deletion.

## Results

### MarvelD3 is expressed in the developing pancreas

**Figure 1:**
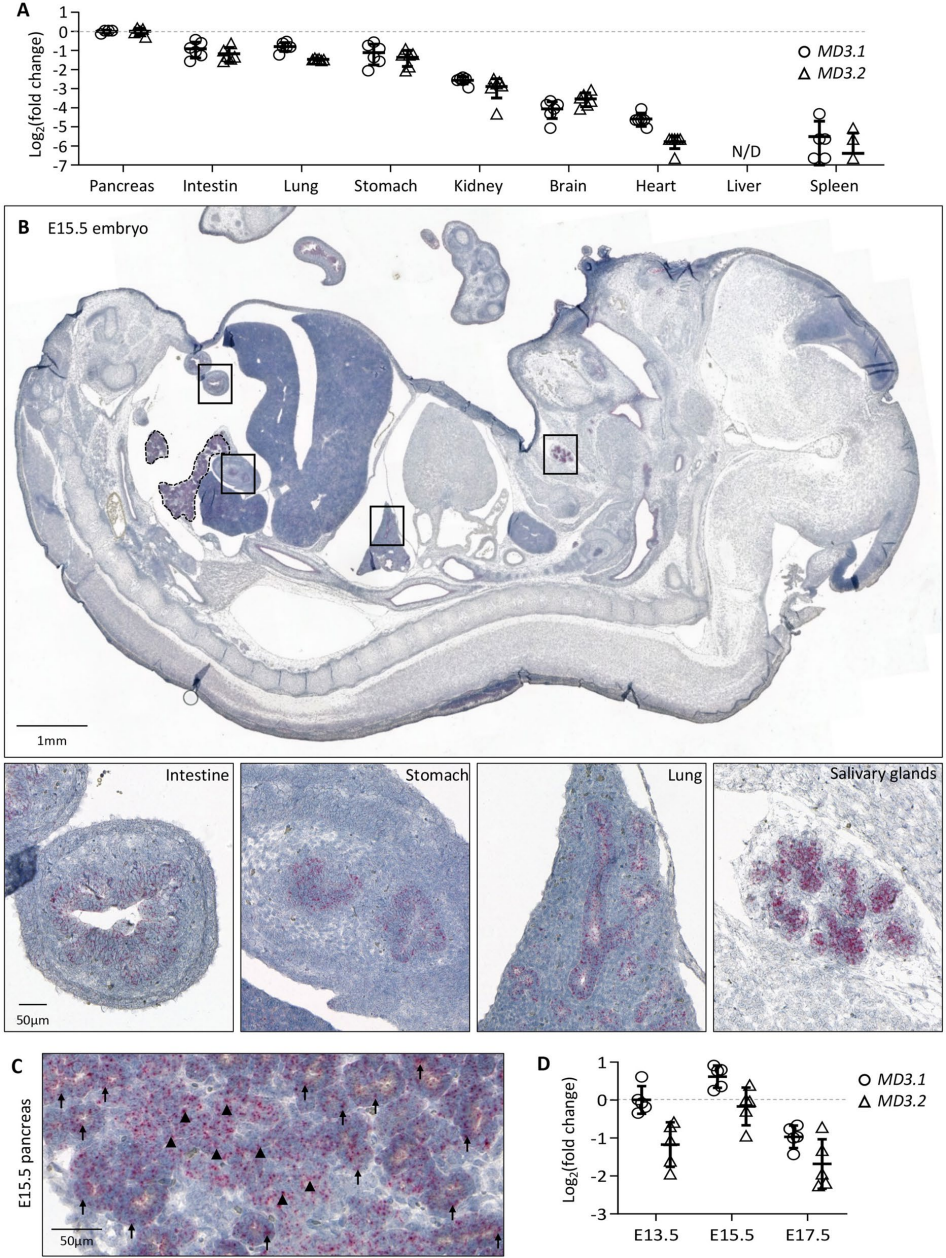
Expression of *MarvelD3* in developing mouse embryo. (**A**) Expression levels of both isoforms of *MarvelD3* (*MD3.1*, open circle; and *MD3.2*, open triangle), measured by RT-qPCR, in epithelial (pancreas, intestine, lung, stomach, kidney, liver) and non-epithelial (brain, heart, spleen) tissues of developing mouse embryos at day 15.5 of development. *Rpl27* was used as internal PCR control (ΔCt) and the values were then compared to the expression of *MD3.1* and *MD3.2* in the embryonic pancreas (ΔΔCt) since it displayed the highest expression level. Data are represented in Log_2_ fold changes. (**B**) Localisation of *MarvelD3 *mRNA (pink dots) by RNAScope in situ hybridization on a sagittal haematoxylin-counter stained section of an E15.5 mouse embryo. *MarvelD3* mRNA is detected in the intestine, stomach, lung and salivary glands (boxes, and high magnifications). Dotted line delineates the pancreas. (**C**) *MarvelD3* mRNA detection in the E15.5 pancreas. The MarvelD3 probe hybridizes with all the cells of the branched epithelial tissue. Acinar structures are indicated with arrows, and ductal structures with arrowheads. (**D**) Expression of *MD3.1* (open circle) and *MD3.2* (open triangle) at three embryonic stages (E13.5-E15.5-E17.5) of pancreas development. Isoform *MD3.1* presents higher expression levels than isoform *MD3.2* throughout development. *β-actin* was used as internal PCR control (ΔCt) and the values were then compared to the expression of *MD3.1* at E13.5 (ΔΔCt). Data are represented in Log_2_ fold changes. Expression levels of *MarvelD3* increase from E13.5 to E15.5, then decrease at E17.5.

Expression of the TJ protein MarvelD3 has been reported in several epithelial cell lines^15^ and adult murine epithelial tissues^14,15^. Here, we first verified that MarvelD3 is expressed in vivo, in epithelial organs of mouse embryos at day 15.5 of embryonic (E) development (E15.5) (Fig. 1A). We therefore micro-dissected epithelial (pancreas, intestine, lung, stomach, kidney and liver) and non-epithelial tissues (brain, heart, spleen), and measured the expression of both isoforms of MarvelD3 (MD3.1, open circle and MD3.2, open triangle) by real-time quantitative RT-qPCR (Fig. 1A). The gene coding for ribosomal protein Rpl27 was chosen as housekeeping gene (HKG), as its expression was similar in all embryonic tissues. Since the ΔCt between *MarvelD3 (MD3.1* and *MD3.2*) and *Rpl27* was the lowest in the pancreas, we considered the pancreas as the tissue in which expression of *MD3.1* and *MD3.2* was maximal. Expression levels of *MD3.1* and *MD3.2* in the other organs were represented relative to the pancreatic expression levels (set as 0 in Log_2_ scale, Fig. 1A). The intestine, lung, stomach and kidney express *MD3.1* and *MD3.2*, although to a lesser extent than the pancreas (respectively 53%, 58%, 46% and 17% for *MD3.1*, and 44%, 36%, 39% and 13% for *MD3.2*). On the contrary, expression of *MarvelD3* could not be detected in the liver at the stage analysed. In non-epithelial organs, *MarvelD3* expression levels were low, and could be attributed to the expression of *MarvelD3* in the blood vascular network of these organs. We then used the RNAScope in situ hybridization technique to localize the cell types expressing *MarvelD3* mRNAs on E15.5 embryo sections (Fig. 1B). Sagittal paraffin sections were incubated with probes targeting a sequence common to both isoforms of *MarvelD3*. The E15.5 embryo showed pinkish dots standing out from the haematoxylin-stained tissues in epithelial organs such as the intestine, the stomach, the lung, the salivary glands (Fig. 1B, boxes from left to right) and the pancreas (Fig. 1B, dotted line and Fig. 1C). At higher magnification (Fig. 1C and S1A), we observed a strong signal in the developing branched structures that correspond to the developing pancreatic acini (arrows) and ducts (arrowheads). Based on the expression pattern of *MarvelD3* in *Xenopus laevis*^17^, we also investigated whether *MarvelD3* was expressed in tissues deriving from the ectodermal sheet, in mouse. We found hybridization of the *MarvelD3* probe in the skin and several parts of the central nervous system such as the pituitary gland (anterior and posterior)^24^ and the choroid plexus (Figure S2). Finally, we monitored the mRNA levels of *MarvelD3* isoforms by RT-qPCR during pancreas development (Fig. 1D), and found that their expression reached a maximum at E15.5, followed by a decrease at E17.5. At all stages examined, isoform *MD3.1* presented a higher expression level than *MD3.2*. Together, these data show that *MarvelD3* is expressed in several developing epithelial tissues with a highest expression level in the developing pancreatic epithelium.

### Generation and validation of a CRISPR-Cas9 MarvelD3 knockout mouse

**Figure 2:**
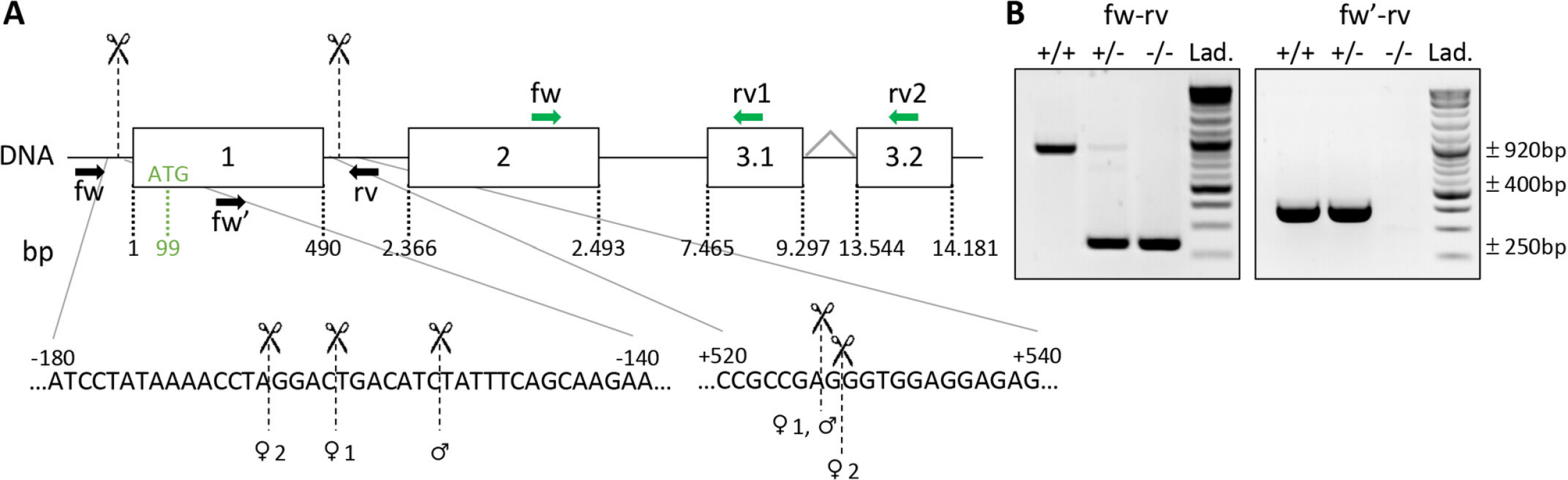
CRISPR/Cas9-based editing of *MarvelD3* gene. (**A**) Schematic representation of the *MarvelD3* locus. The *MarvelD3* gene comprises four exons (boxes), the two first are common while either exon 3.1 or 3.2 are alternatively spliced to generate the two isoforms (MD3.1 and MD3.2). Numbers below the boxes indicate the position of the exons limits with respect to the transcription initiation site (+ 1). Position of the ATG (99 bases downstream of the + 1) in the first exon is also indicated. Scissors and the vertical dashed lines indicate the approximate region where the cuts occurred. Precise localization of the cuts is provided below for the three individuals used to derive the colony. Localisation of genotyping primers are represented by black arrows (fw-rv and fw′-rv); primers to assess *MarvelD3* expression by RT-qPCR are shown in green (fw-rv1 = *MD3.1* and fw-rv2 = *MD3.2*). (**B**) Illustrative genotyping results for wild-type (+ / +), heterozygous (+ /−) and knockout (−/−) mice, obtained with two sets of primers. Primers fw-rv, located outside of the targeted region (exon 1), can generate PCR products of ± 920 bp (wild-type allele: +), and ± 250 bp (deleted allele: -). Primers fw′-rv will only amplify a band of ± 400 bp on the wild-type allele (+ / + and + /−). Lad., DNA ladder.

Knock-down of MarvelD3 in *Xenopus laevis* embryos results in developmental defects of ectodermal derivatives^16,17^. In mice, we also detected *MarvelD3* mRNA in tissues of ectodermal origin (Figure S2), but it was predominantly observed in endoderm-derived epithelial organs such as the pancreas. To study the in vivo role of MarvelD3 in the pancreas, we generated a MarvelD3^−/−^ model using a CRISPR-Cas9 gene editing system with two guide RNAs (Fig. 2A). This allowed removal of a DNA fragment of approximately 685 bp, encompassing the transcription initiation site, the first exon and part of the first intron. Excision of this fragment, common to both MarvelD3 isoforms, was verified by PCR (Fig. 2B). Amplicons generated with primers located outside of the targeted regions (fw-rv), gave rise to a ± 920 bp band for the wild-type allele and a ± 250 bp band for the deleted allele (Fig. 2B, left). Amplification of the wild-type allele in heterozygous (+ /−) mice was weaker than in homozygous (+ / +). A second forward primer (fw′), located within the first exon, was also used with the reverse primer (rv) (Fig. 2B, right). Amplification of a ± 400 bp band revealed the presence of at least one wild-type allele (in + / + and + /−), while in knockout mice (−/−) no amplification was possible as the forward primer (fw′) was in the deleted region. Finally, Sanger sequencing of the ± 250 bp amplicons revealed that the extent of the deletion ranged from 679 to 691 bp in the three founders used to generate the colony (Fig. 2B).

### MarvelD3 is dispensable for normal pancreas development and morphology

MarvelD3 knockout embryos were obtained at the expected Mendelian ratio. Moreover, knockouts were indistinguishable from control littermates and macroscopic observation did not reveal any morphological defects (data not shown). Adult knockout mice of both genders were viable (> 1 year) and fertile.

**Figure 3:**
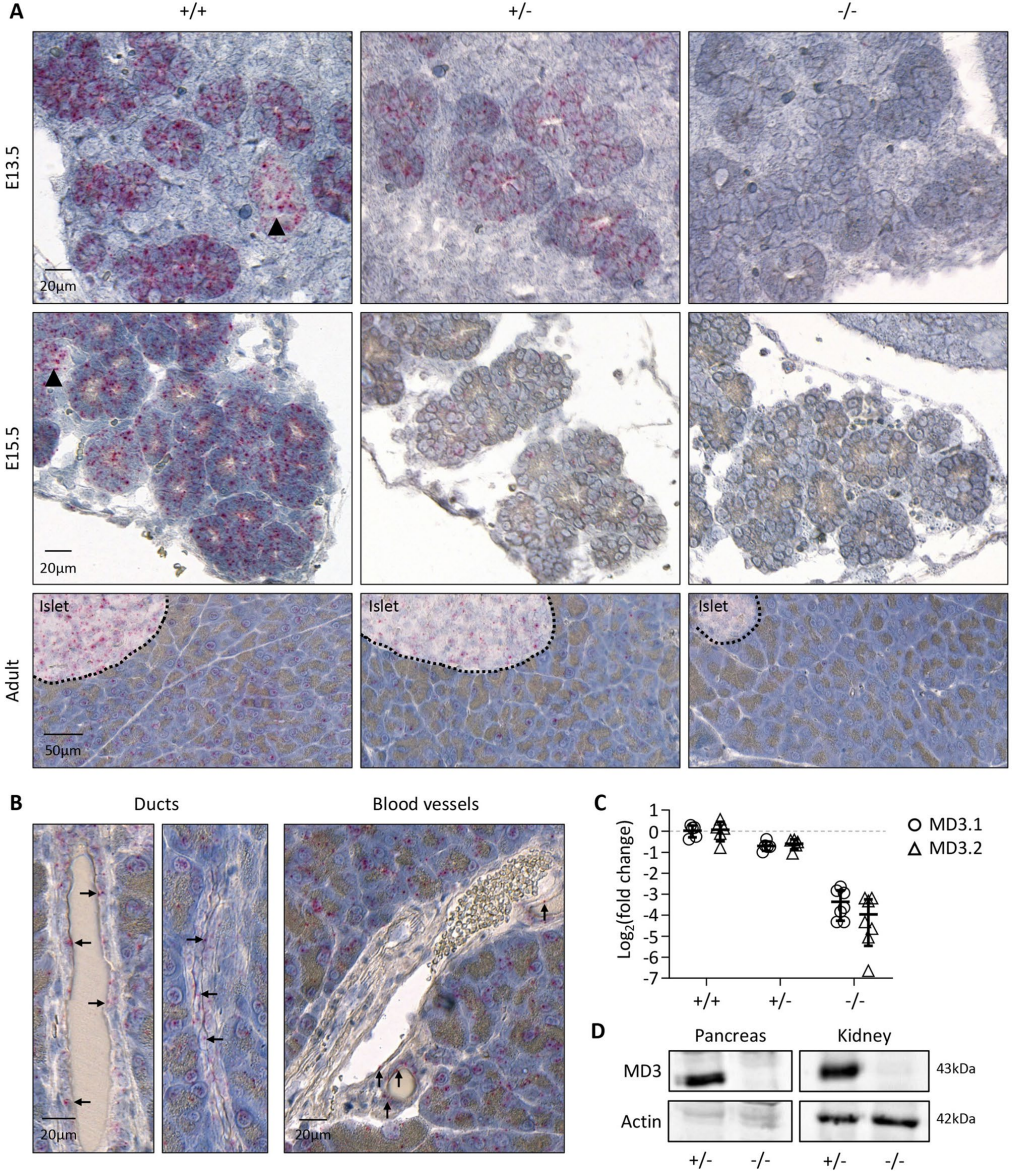
Validation of the *MarvelD3* knockout mouse model. (**A**) Detection of *MarvelD3* mRNA by RNAScope in situ hybridization on embryonic (E13.5 and E15.5) and adult pancreata from wild-type (+ / +), heterozygous (+ /−) and knockout (−/−) mice. Fast red chromogenic signals (pink dots) obtained with the *MarvelD3* probe in wild-type tissues illustrate normal *MarvelD3* mRNA expression. Deletion of one allele generates intermediate signal intensity, while deletion of the two *MarvelD3* alleles abolishes pink staining. (**B**) High magnification images of *MarvelD3* mRNA by RNAScope in situ hybridization in wild-type adult pancreas. *MarvelD3* mRNA (pink dots) is found in the flattened intercalated and cuboidal intralobular pancreatic ductal cells (arrows), and in endothelial cells (arrows) of blood vessels of different sizes, located within the pancreatic parenchyma. (**C**) Relative expression levels of *MarvelD3* isoforms (*MD3.1*, open circle; *MD3.2*, open triangle) measured by RT-qPCR in E15.5 pancreata of + / + , + /− and −/− embryos. An approximate 50% and 90% decrease in *MarvelD3* expression was observed in + /− and −/− mice, respectively, as compared to wild-type (+ / +) mice. *β-actin* was used as internal PCR control (ΔCt) and the values were then compared to the expression of *MD3.1* or *MD3.2* in the + / + (ΔΔCt). Data are represented in Log_2_ fold changes. (**D**) Western blotting of MarvelD3 (MD3) and β-actin (Actin) in pancreatic and renal protein lysates of heterozygous (+ /−) and knockout (−/−) mice. MarvelD3 can be detected in heterozygous pancreas and kidney, while it is absent in knockout tissues. Actin levels vary between tissues, but are constant in the different samples of the same tissue. Full-length blots/gels are presented in Supplementary Fig. 6.

As *MarvelD3* expression levels were the highest in the pancreas (Fig. 1A), we investigated the impact of MarvelD3 deletion on pancreas development, at tissular level. Therefore, we collected pancreata from wild-type (+ / +), heterozygous (+ /−) and knockout (−/−) mice at different embryonic stages (E13.5 and E15.5) and at adulthood. First, we performed RNAScope in situ hybridization of *MarvelD3* on pancreas sections (Fig. 3A). Wild-type embryonic pancreatic tissues displayed a strong staining for the *MarvelD3* probe in all the epithelial cells, indicating that *MarvelD3* is highly expressed in the embryonic pancreatic parenchyma (Fig. 3A, left panels). The surrounding connective tissue was not stained by the *MarvelD3* probe. In adult wild-type tissues, *MarvelD3* was detected in acinar cells and endocrine cells of the islets of Langerhans (Fig. 3A, lower panel). Pancreatic ducts and blood vessels were not visible on that section. However, at higher magnification (Fig. 3B), we observed that epithelial cells of the intercalated (flattened epithelium, left) and intralobular (cuboidal epithelium, right) ducts, and endothelial cells of blood vessels were also positively stained by the *MarvelD3* probe (arrows). Thus, *MarvelD3* seems to be expressed in the exocrine and endocrine compartments of the pancreas in embryos and in adults. Pancreata with deletion of one *MarvelD3* allele (+ /−) presented a similar, but weaker, staining pattern at all stages of development and in the adult pancreas (Fig. 3A, middle panels). On the contrary, the *MarvelD3* probe did not react with the knockout tissues (Fig. 3A, right panels), thereby validating *MarvelD3* inactivation. Interestingly, the size and the general architecture of the pancreas, highlighted by the haematoxylin staining, was indistinguishable between wild-type, heterozygous and knockout pancreata at all stages. We then performed RT-qPCR for *MarvelD3* isoforms on RNA extracted from pancreata of the three genotypes (Fig. 3C). As compared to wild-type, which displayed high expression levels of isoforms *MD3.1* (open circle) and *MD3.2* (open triangle) in the pancreas at E15.5, heterozygous and knockout pancreata showed a reduction of almost 50% and 90% in the expression of both isoform-coding mRNAs, respectively. This indicate that although the transcription initiation site was deleted in our knockout allele (Fig. 2A), an illegitimate transcript could be produced at low rate since an amplicon encompassing part of exon 2 and of exon 3 could be generated. The abundance of this transcript was approximately tenfold lower, as compared to the wild-type transcript (Fig. 3C). Finally, western blotting analyses on adult pancreatic and renal protein extracts revealed the presence of a 43 kDa band in heterozygous tissues, which was undetectable in knockout organs (Fig. 3D). The CRISPR-Cas9 MarvelD3 inactivation thus lead to a complete absence of MarvelD3 full-length protein.

### Pancreas differentiation is preserved in MarvelD3 knockouts

We have seen that MarvelD3 knockouts are viable and fertile, and that pancreas size and general morphology are not affected by MarvelD3 deletion. We next investigated whether the absence of MarvelD3 impacts embryonic cellular differentiation and/or function of the adult pancreas.

**Figure 4:**
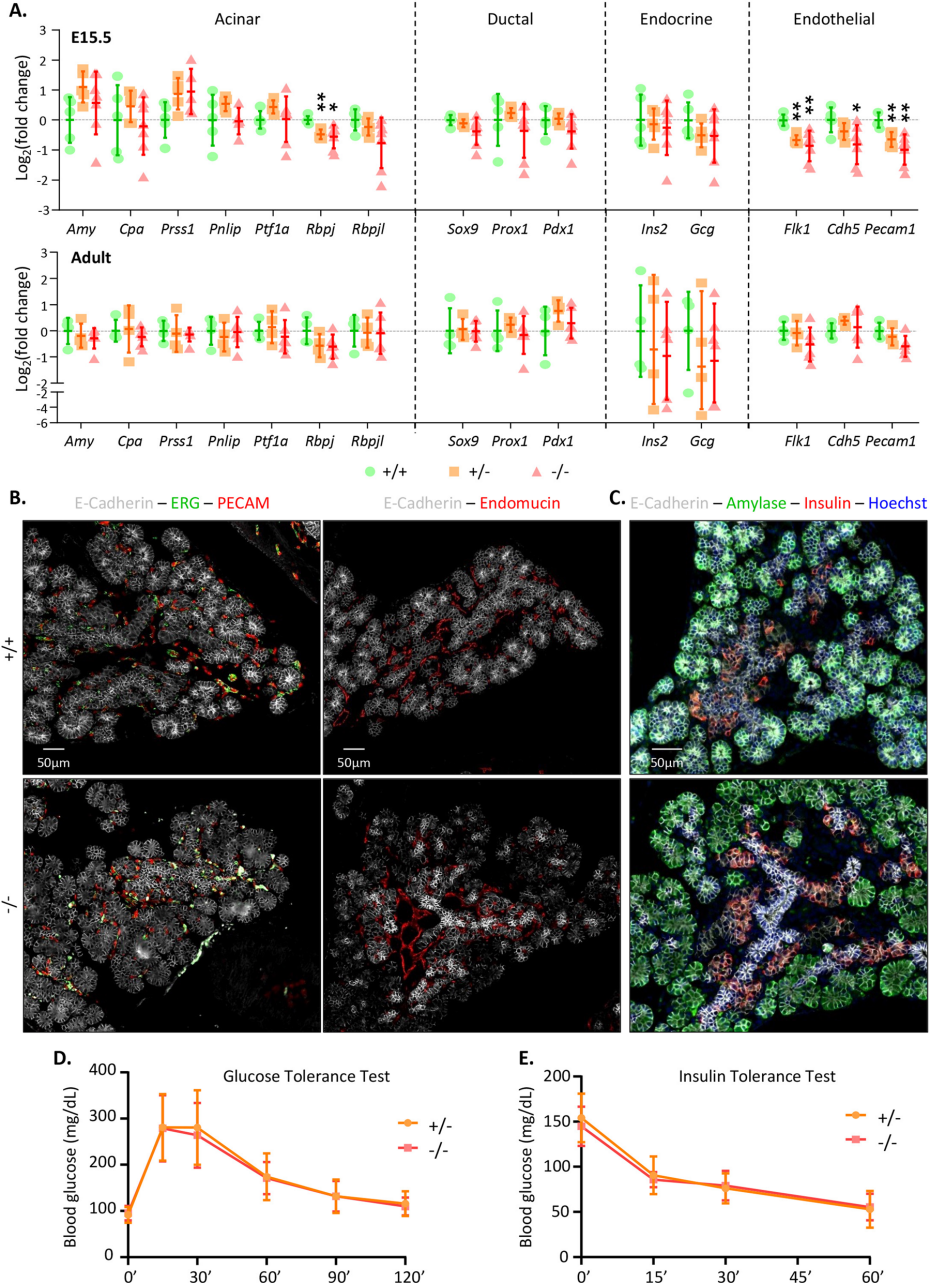
Absence of MarvelD3 does not impair pancreas differentiation and function. (**A**) Expression levels of acinar, ductal, endocrine and endothelial genes measured by RT-qPCR in wild-type (+ / + , green circles), heterozygous (+ /−, orange squares) and MarvelD3 knockout (−/−, red triangles) pancreata at E15.5 and adulthood. *β-actin* was used as internal PCR control (ΔCt) and the values were then compared to the expression of the + / + pancreata (ΔΔCt). Data are represented in Log_2_ fold changes. Downregulation of *Rbpj*, *Flk1*, *Cdh5* and *Pecam1* is observed in E15.5 −/− pancreata, compared to + / + pancreata. All other gene expression levels are comparable between the three genotypes. No gene expression changes can be observed in adult tissues. Statistically significant Mann–Whitney test with *p*-value < 0.05 (*), and *p*-value < 0.01 (**). (**B**) Identification of endothelial and epithelial cells by immunostaining for the markers ERG (green), PECAM (red), Endomucin (red), and E-Cadherin (white), respectively, on E15.5 wild-type (+ / +) and knockout (−/−) pancreas sections. Endothelial cells, with an ERG^+^ nucleus (green) and either a PECAM^+^ (left) or Endomucin^+^ (right) cytoplasm (red), are predominantly located around the central epithelial (E-Cadherin, white) cells. Their number and localisation are similar in + / + and −/− tissues. (**C**) Immunolabeling of the exocrine marker amylase (green) and endocrine marker insulin (red) within the pancreatic E15.5 epithelium (E-cadherin, white) of + / + and −/− mice. Both tissues show peripheral ovoid structures expressing amylase (green), and groups of insulin^+^ (red) cells close to the central epithelial branches. (**D**) Glucose and (**E**) insulin tolerance test of MarvelD3 + /− and −/− mice. Two- and four-months old males and females, either heterozygous or knockout for MarvelD3 were injected with glucose or insulin solution, and blood glucose levels (in mg/dL) were monitored along time. Hypoglycaemia or hyperglycaemia measurements are not impacted by MarvelD3 deletion.

We first measured the expression of various markers of the different MarvelD3-expressing cell types of the pancreas, by RT-qPCR (Fig. 4A). Expression of the genes coding for digestive enzymes (amylase, carboxypeptidase A, trypsin, lipase), acinar transcription factors (Ptf1a, Rbpj, Rbpjl), ductal/embryonic markers (Sox9, Prox1, Pdx1), endocrine hormones (insulin, glucagon) and endothelial markers (Vegfr2/Flk1, VE-Cadherin, Pecam1) was assessed in wild-type (+ / + , green circles), heterozygous (+ /−, orange squares) and MarvelD3 knockout (−/−, red triangles) pancreata at E15.5 and at adulthood (Fig. 4A). Expression of all these markers, but *Rbpj* and the three endothelial markers, were comparable between embryonic wild-type and knockout tissues. In adult, no statistical differences were observed. Rbpj is an embryonic transcription factor that forms the transcriptional PTF1-J complex in association with Ptf1a, which regulates pancreas growth and morphogenesis^25^. Around E14.5, Rbpj is replaced by Rbpjl in the transcriptional complex, and its expression starts to decrease^25^. Therefore, a decrease in *Rbpj* expression could indicate a premature initiation of the acinar differentiation process. However, expression levels of the pro-acinar transcription factors *Ptf1a* and *Rbpjl* were not increased in the knockout tissue, and immunolabeling of amylase-expressing cells at E13.5 did not reveal premature acinar differentiation in the MarvelD3 knockout tissue (Figure S1B). Moreover, as the decreased *Rbpj* expression level in MarvelD3 knockout was observed after induction of acinar differentiation, we did not further investigate its expression levels. The expression of three genes from the endothelial lineage was also affected in MarvelD3 knockouts. As endothelial cells have been shown to regulate acinar differentiation^26,27^, we further analysed the endothelial compartment by multiple immunolabeling on E15.5 pancreas sections (Fig. 4B). We used antibodies directed against the endothelial transcription factor ERG (green) and the endothelial cell adhesion molecule PECAM (red), together with the epithelial cadherin, E-cadherin (white) (Fig. 4B, left). We also stained pancreatic sections for the endothelial glycoprotein endomucin (red) to visualize the network of blood vessels and the pancreatic epithelium (E-cadherin, white) (Fig. 4B, right). Despite gene expression changes (Fig. 4A), no differences were observed in localisation, abundance and general organization of blood vessels in MarvelD3 knockouts (Fig. 4B). Finally, qualitative analysis of exocrine and endocrine differentiation was assessed by immunolabeling of amylase (green) and insulin (red) respectively, in wild-type (+ / +) and MarvelD3 knockout (−/−) pancreata. As differentiation is initiated at E14.5, we continued our analysis at E15.5. Knockout tissues presented a similar expression pattern and intensity for the acinar and the endocrine markers, as wild-type controls (Fig. 4C). This was confirmed by quantifications using an image software analysis (Figure S3A). We concluded that the absence of MarvelD3 does not impair, nor delays appearance of these two differentiation markers.

As exocrine and endocrine differentiation were not affected by MarvelD3 deletion, we next investigated the function of both compartments in adult mice. The exocrine function of the pancreas was first analysed by assessing the abundance of the acinar protein amylase, and of the ductal transcription factor Sox9 by western blotting. No difference was observed in MarvelD3 knockout pancreata, as compared to control (Figure S3B,C). We then indirectly evaluated the exocrine function by controlling the mice’s weight, analysing the morphology and histology of their digestive system, and by monitoring the aspect and quantity of their faeces (Figure S4). Based on these criteria, we concluded that the exocrine function of the pancreas was not affected in the absence of MarvelD3. Endocrine function of the pancreas was directly assessed by a glucose tolerance test (GTT; Fig. 4D). We also evaluated glucose clearance in peripheral tissue by an insulin tolerance test (ITT; Fig. 4E). Since glucose metabolism can be impacted by age and gender^28^, we performed GTT and ITT in heterozygous (+ /−) and knockout (−/−) males and females, at 2 and 4 months of age. Glycaemia was measured before glucose injection, then at 15, 30 and 60 min during the first hour and finally at 90 and 120 min. After 2 h, glycaemia returned to its initial value. Transient upregulation and subsequent normalization of the blood glucose levels after glucose injection were similar in heterozygous and knockout mice (Fig. 4D). For the ITT (Fig. 4E), glycaemia was also measured prior to insulin injection (0′), and then evaluated every 15 min (15′, 30′, 60′) (Fig. 4E). Due to severe hypoglycaemia, the ITT was then terminated by glucose injection. The linear decrease of blood glucose levels upon insulin injection were comparable between heterozygous and knockout mice of all genders and ages. Results are thus represented as a mean of the four groups (males and females, at 2 or 4 months of age). Thus, pancreatic functions, i.e. food digestion and blood glucose homeostasis, were also unaffected following MarvelD3 deletion.

### Normal pancreatic epithelial polarity, without compensatory upregulation or relocation of other TAMPs, in the absence of MarvelD3

Tight junction proteins have been shown to participate in cell polarity and in the control of several biological processes through interactions with signalling pathways^29^.

**Figure 5:**
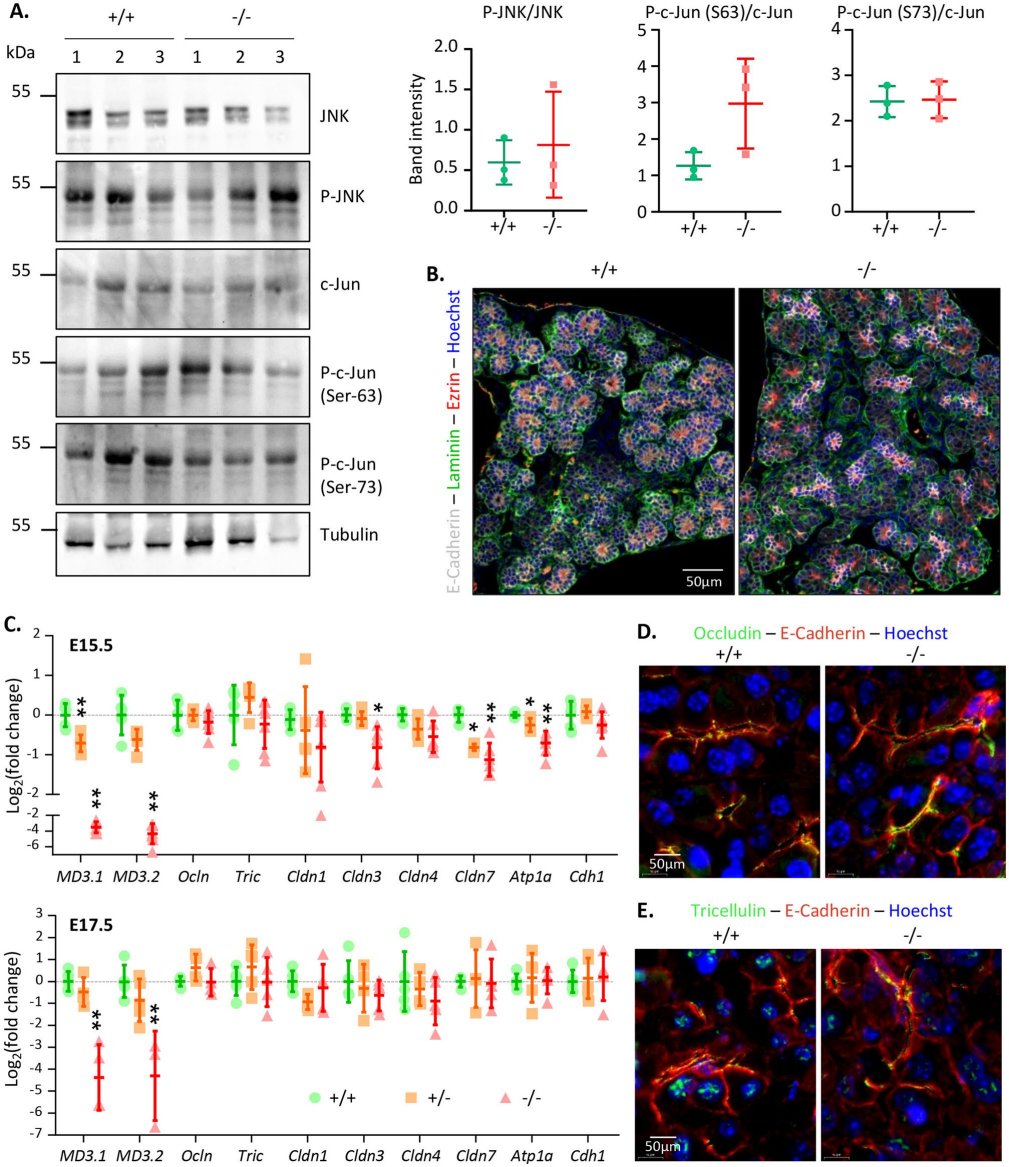
JNK-pathway activity and cell polarity are not altered in the absence of MarvelD3. (**A**) Detection of JNK, c-Jun and their phosphorylated forms in protein extracts from 3 wild-type (+ / +) and 3 MarvelD3 knockout (−/−) pancreata by western blotting. No differences in JNK, phospho-JNK (P-JNK), c-Jun and phospho-c-Jun (P–c-Jun, Ser63 and Ser73) protein levels were observed between wild-type and knockout pancreata. Quantification of the bands (P-JNK/JNK and P–c-Jun/c-Jun) is shown on the right. Full-length blots/gels are presented in Supplementary Fig. 8. (**B**) Cell polarity assessment by immunolabeling of apical (ezrin, red) and basal (laminin, green) markers of epithelial (E-Cadherin, white) cells on E15.5 embryonic pancreas sections. Wild-type (+ / +) and knockout (−/−) pancreatic epithelial cells are polarized. (**C**) RT-qPCR evaluation of tight junction and baso-lateral marker gene expression levels in embryonic (E15.5 and E17.5) pancreata of wild-type (+ / + , green circles), heterozygous (+ /−, orange squares) and MarvelD3 knockout (−/−, red triangles) mice. Transient downregulation of *claudin-3* and *-7*, and *NaK-ATPase* (*Atp1a*) is observed in E15.5 knockouts. (**D–E**) Immunolabeling of the TAMP occludin and tricellulin (green) in the wild-type and knockout E15.5 pancreatic epithelium (E-Cadherin, red). The absence of MarvelD3 does not trigger relocalization of the two other TAMPs.

MarvelD3 can interact with the MAPK kinase kinase MEKK1 to regulate the activity of the JNK-pathway^16,17,30^. We thus investigated whether the absence of MarvelD3 triggers changes in JNK expression levels and/or activation (evaluated by phospho-JNK) (Fig. 5A). We also analysed the abundance and phosphorylation of c-Jun, a target of JNK. Three control and three knockout pancreata were analysed by western blotting and the intensity of the bands was quantified. We could not detect differences in the intensity of total- and phospho-protein levels in wild-type (+ / +), and knockout (−/−) pancreas samples. Thus, in adult pancreata the activity of the JNK-pathway does not seem to be affected by the absence of MarvelD3.

In vitro observations revealed that MarvelD3 does not participate in polarity establishment, but is required for regulation of paracellular permeability^15^. However, the potential role of MarvelD3 in vivo in mice was unknown. We thus assessed cell polarity in wild-type (+ / +) and knockout (−/−) pancreata by immunolabeling of the apical marker Ezrin (Fig. 5B, red) and the basal marker Laminin (green). Co-immunolabeling of the epithelial cadherin (E-Cadherin in white) revealed that the branched structure of developing pancreas (i.e. ducts terminated by acini) were delineated by a laminin-containing basement membrane (green). The narrow acinar and ductal lumina were visualized in red (Ezrin). Wild-type and knockout tissues presented a similar labelling pattern, and apico-basal polarity, as assessed by these two markers, was normal in the pancreatic epithelium lacking MarvelD3. We thus confirmed that polarization of pancreatic epithelial cells in vivo can occur in the absence of MarvelD3.

Assessment of paracellular permeability of the pancreatic epithelium is difficult to realize in embryos. We nevertheless excluded the presence of inflammatory infiltrates in the stomach of one-year old MarvelD3 knockout embryos (Figure S5), thereby suggesting unaltered paracellular permeability^11^. In addition, tissue histology and exocrine function/food digestion were not affected in the absence of MarvelD3 (data not shown).

It has been shown that the absence of a protein can be compensated by upregulation of proteins with similar functions and/or domain redundancy^31^ or by relocalization of proteins. This has been observed for tight junction proteins. The phenotype of the occludin knockout mice is partially explained by the relocalization of tricellulin (MarvelD2) from tricellular to bicellular junctions^32,33^. We thus investigated whether the absence of phenotype in the pancreas of MarvelD3 knockouts could be explained by alteration of TAMP expression levels or patterns. RT-qPCR experiments confirmed a ± 50% and > 90% downregulation of *MD3.1* and *MD3.2* in heterozygous (+ /−, orange squares) and knockouts (−/−, red triangles), respectively, as compared to wild-type (+ / + , green circle) embryos at E15.5 and E17.5 (Fig. 5C). Expression of *Ocln*, and *Tric* (*MarvelD2*) was comparable in the three genotypes, thereby excluding compensatory upregulation of other TAMP members in the absence of MarvelD3. We also measured the expression levels of claudins reported to be expressed in the embryonic pancreas epithelium^34^, namely *claudin-1*, *-3*, *-4* and *-7*. While reduction of *claudin-7* expression levels was already observed in E15.5 heterozygous tissues, downregulation of *claudin-3* was only observed in knockout tissues. Although these reductions were statistically significant, they were only transient, as normalized expression levels were already obtained at E17.5, and could not compensate for the loss of MarvelD3. Gene expression of other claudins was not altered in the absence of MarvelD3. Assessment of the expression of baso-lateral markers (NaK-ATPase and E-Cadherin) further revealed that epithelial cells lacking MarvelD3 presented lower expression levels of the NaK-ATPase (*Atp1a*) at E15.5. This effect was also transient, as expression levels were normalized two days later. On the contrary, E-Cadherin (*Cdh1*) expression was not impacted by MarvelD3 deletion. In conclusion, although expression of some junctional markers was decreased in knockout pancreata, no compensatory upregulation of tight or adherens junction proteins could be measured.

Potential relocalization of the TAMP proteins in MarvelD3 knockout pancreata was finally evaluated by immunolabeling of occludin (green, Fig. 5D) and MarvelD2 (tricellulin, green, Fig. 5E), within the pancreatic epithelium (E-Cadherin, red). Occludin was found at the apical membrane, opposing the basally located nucleus (Hoechst^+^, blue) of wild-type pancreatic epithelial cells. In the absence of MarvelD3, its expression pattern was not altered. The localisation of tricellulin in the wild-type pancreas was both membranous, at the apical pole, and nuclear. The knockout tissue presented a similar localization pattern.

To conclude, establishment of cell polarity in the embryonic pancreas and its maintenance in the adult tissue is not affected by MarvelD3 deletion. Moreover, the absence of MarvelD3 is not compensated by upregulation or relocalization of the most obvious tight and adherens junction proteins.

## Discussion

In this work we established the spatial expression pattern of the tight junction protein MarvelD3 in the developing mouse embryo, and found that *MarvelD3* is expressed in several epithelial tissues, and particularly abundant in the pancreas. Since all pancreatic cell types, namely acinar, ductal and endocrine cells, but also endothelial cells displayed positive staining for *MarvelD3* mRNA by RNAScope in situ hybridization, we investigated whether pancreas histology, differentiation and function were altered upon *MarvelD3* genetic ablation. We did not observe any pancreatic phenotype in the MarvelD3 knockout mice, at embryonic or adult stages.

Other groups reported *MarvelD3* expression in epithelial and endothelial cell lines, and in adult epithelial tissues^14,15^. Here, we showed that *MarvelD3* is already expressed in the developing pancreas from day 13.5 of embryonic development (E13.5), and in other epithelial tissues such as the salivary glands, at E15.5. It was striking to observe that the two most reactive tissues for the *MarvelD3* probe were the pancreas and the salivary glands. Histologically and functionally, these two glandular organs present similarities as they are specialized in the secretion of a bicarbonate-rich fluid containing digestive enzymes^35^. It has been shown that the secretory function of both organs is altered in IgG4-related disease (IgG4RD), a chronic inflammatory pathology. Reports have established a correlation between the expression levels of tight junction proteins and their specific localisation at the apical tight junction, and proper secretory function of pancreas and salivary gland^36^. Therefore, a role for MarvelD3 could be found in this particular pathological condition in these two glands.

Our data also supported the previously observed differential expression levels of both MarvelD3 isoforms depending on the tissue^14^. In the pancreas, isoform *MD3.1* presented higher relative expression than *MD3.2* at all stages of development analysed. Moreover, expression of both isoforms increased until E15.5, and then diminished. Dynamic changes in tight junction protein expression levels have been reported in the developing pancreas^34^, and were shown to last until complete maturation of the tissue, around weaning^37^. Since it has been shown that *MarvelD3* mRNA synthesis is directly followed by protein incorporation to the tight junction^14^, we propose that MarvelD3 incorporation to the tight junction would be maximal around E15.5.

To study the role of MarvelD3 in vivo in mice, we generated a total knockout by CRISPR-Cas9-mediated *MarvelD3* gene disruption. The chosen strategy relied on the use of two gRNAs to increase the chances of removing a large DNA sequence (i.e. the interval between the two gRNAs) in the gene of interest, which can be easily detected by classical genotyping PCR^38^. The position of the gRNAs was chosen to avoid initiation of transcription and to prevent production of both MarvelD3 isoforms (N-terminal sequence is common). Indeed, accumulation of mRNAs with a premature termination codon can trigger compensatory mechanisms that could mask the MarvelD3-specific phenotype^39^. Note that before embarking in the CRISPR-Cas-mediated *MarvelD3* disruption, we first analysed total and pancreas-specific KO for MarvelD3 obtained with a classical Cre-LoxP strategy, i.e. PGK-Cre or Pdx-Cre mice crossed with mice bearing the KOMP *MarvelD3* floxed allele (Marveld3^tm45743(L1L2_Bact_P)^). Although similar, these results were not presented since we found that upon Cre-dependent deletion of the floxed *MarvelD3* exon 2, the KOMP construct generated a mRNA encompassing exon 1 and MarvelD3 ATG, and possibly a truncated protein (data not shown).

Developmental defects of tissues of ectodermal, but not of endodermal origin have been described in the *Xenopus laevis* MarvelD3 morphants^16,17^. Based on the mouse embryo expression pattern, we focused on the endodermal-derived pancreas and assessed if the pancreatic tissue would be structurally and functionally affected by MarvelD3 deletion. The size, the shape and the general architecture of knockout embryonic and adult pancreata were indistinguishable from their wild-type counterparts. Differentiation of the pancreas starts around E14.5, a time-point known as the secondary transition. We evaluated whether expression levels of typical markers for each pancreatic compartment were altered upon MarvelD3 deletion, one day after the initiation of differentiation (E15.5). Expression levels of digestives enzymes (amylase, carboxypeptidase A, trypsin and lipase) and of hormones (insulin and glucagon) were highly variable in E15.5 embryos of all three genotypes, but no trend could be observed. On the contrary, expression levels of transcription factors (Ptf1a, Rbpjl, Sox9, Prox1, Pdx1) involved in pancreas differentiation were stable but still comparable between wild-type and MarvelD3 knockouts. These observations indicate that the secondary transition was initiated in all embryos, but that the timing of differentiation could slightly vary between individuals, independently of their genotype, thus resulting in variable enzymatic and hormonal levels. In addition, normal differentiation was confirmed by immunolabeling of amylase- and insulin-expressing cell, accounting for the acinar and endocrine compartments respectively. The only transcription factor that showed a decreased gene expression in MarvelD3 knockout embryos was *Rbpj*. However, as Rbpj is required for early pancreas growth and morphogenesis, which seemed unaltered in E15.5 knockout tissues (i.e. size and architecture were similar to wild-type tissues), we did not further investigate this transient difference. Three markers of the endothelial compartment were down-regulated in E15.5 knockout pancreata. However, the significance of this observation was not understood as multiplex immunolabeling revealed that the localisation, the number and the organization of blood vessels were comparable between wild-type and knockout embryonic tissues. Decreased expression could indicate a delayed differentiation or maturation of endothelial cell-forming vessels. In adult tissues, the expression levels of all analysed genes were comparable in wild-type and knockout mice. The high variability observed in the expression of genes from the endocrine compartment could be explained by the size, and thus the number of islets of Langerhans, of the tissue pieces selected for RT-qPCR experiments. Altogether, the absence of MarvelD3 did not impact the onset and maintenance of pancreatic differentiation. Accordingly, the endocrine and exocrine functions of the pancreas were not affected by MarvelD3 deletion.

Tight junction (TJ) proteins form the backbone of TJs and are required to maintain cell polarity^29^. MarvelD3 knockout embryonic pancreatic cells were polarized, as evidenced by normal apical staining for ezrin and basal staining for laminins. We also investigated whether the absence of MarvelD3 could be compensated by upregulation of genes with similar functions and/or domain redundancy. We therefore assessed the expression of the main TJ proteins (claudin-1, -3, -4 and -7, which are expressed in the pancreas), and of occludin and tricellulin, as they share a similar structural domain (MARVEL domain). A decrease in expression of *claudin-3* and *-7* could be observed at E15.5. However, this decrease was transient and expression levels were normalized two days later, at E17.5. In adult tissues devoid of MarvelD3, no compensatory upregulation of these genes could be observed. Since TAMP have redundant function^14^ and since some defects detected in the occludin knockout mice are due to a relocalization of tricellulin from tricellular to bicellular contacts^32,33^, we also investigated whether relocalization or mislocalization of these proteins occurred in the absence of MarvelD3. Unfortunately, we did not observe any difference in TAMP localization between control and knockout tissues by classical immunofluorescence. Although we did not evaluate the expression of all tight junction proteins, and even though subtle TAMP rearrangements cannot be excluded, our in vivo data are in agreement with those of Steed et al.^15^ demonstrating that MarvelD3 is not required for cell polarity establishment.

TJ proteins can control the activity of signaling pathways^30,40–42^. Previous studies reported the interaction of MarvelD3 with the MAPK kinase kinase MEKK1^16,17,30^. While in vitro experiments first demonstrated that MarvelD3 retains MEKK1 at the apical membrane in order to prevent activation of the JNK-signalling pathway^30^, in vivo results in *Xenopus* later suggested that MarvelD3 activates JNK-signalling during eye development^17^, while it transiently decreases JNK activity in neural crest induction^16^. Thus, MarvelD3 is implicated in the regulation of the JNK-signalling pathway. Assessment of JNK pathway activity by western blotting in adult pancreas did not support a role for MarvelD3 in the control of the JNK pathway. Literature analysis does not highlight a role for this signalling pathway in adult pancreas. However, one study reports a role for JNK activity in embryonic pancreas development^43^, and several others report the implication of JNK activity in pancreatic diseases such as pancreatitis and pancreatic ductal adenocarcinoma^44–47^. Further studies should investigate whether MarvelD3 is implicated in these pathological conditions.

Based on the high MarvelD3 expression levels in embryonic pancreatic tissue, and based on the developmental defects observed in MarvelD3 *Xenopus laevis* morphants, we were disconcerted by the lack of phenotype in our model. We thus hypothesize that knocking out MarvelD3 could eventually produce a phenotype in a small proportion of the population that we either overlooked, or that would necessitate the analysis of a large number of animals. Another possibility would be that deletion of MarvelD3 could induce an unpredictable effect in tissues/organs that have not been examined, or in (pathological) conditions that have not yet been studied. Indeed, it has been reported that knockout mice for the TJ protein claudin-4 do not present a phenotype under normal conditions, but show a greater degree of lung injury when exposed to hyperoxia^48^. Since MarvelD3 is highly expressed in the pancreas, it would be interesting to assess its role in mice subjected to pancreatitis and/or pancreatic ductal adenocarcinoma (PDAC). This is also supported by the implication of JNK in those diseases^44–47^, and by reports suggesting a role for MarvelD3 when tissue homeostasis is impaired^30^. Indeed, MarvelD3 is necessary to regulate, and prevent, prolonged JNK activity that is known to cause extensive cell death upon induction of hyperosmotic stress. Moreover, siRNA-mediated depletion of MarvelD3 in Caco-2 cells subjected to osmotic stress triggers alterations of the TJ integrity and barrier function^30^. Other groups demonstrated the implication of TJ proteins in pancreatic ductal adenocarcinoma development. First, a reduced expression of several claudins is observed in precancerous PanIN lesions^34^, and expression levels of claudin-7 are correlated with patient survival^49^. In pancreatic ductal adenocarcinoma, the localization of the TAMP protein, tricellulin, is changed to the nucleus and could contribute to cancer progression^41^. The expression level of *MarvelD3* has also been correlated with the differentiation status of pancreatic cancer cell lines^30,50^, and with their ability to migrate, proliferate and form tumours^30^. Finally, Snail-induced epithelial-to-mesenchymal-transition of HPAC cells, a pancreatic cancer cell line, is accompanied by a decrease in *MarvelD﻿3* expression^50^. These data suggest that MarvelD3 deletion could affect the development of pancreatic ductal adenocarcinoma in vivo in mice, and support further studies in that direction. A last hypothesis would be that the deficiency of MarvelD3 would be compensated by occludin and/or tricellulin without affecting their expression levels in these pathological conditions. Several reports indeed indicate that TAMP can modulate the morphology of TJ strands^18,51,52^.

In this study, we report that MarvelD3 is expressed in all epithelial cell types of the developing pancreas, and in several other epithelial organs. Despite the high MarvelD3 expression levels in the pancreas, we did not observe alterations of pancreas development, histology and function upon genetic ablation of MarvelD3 in mice with a homogeneous genetic background, raised in a protected environment.

## Materials and methods

### Animals

MarvelD3 knockout mice (MD3^−/−^) were generated by the CRISPR-Cas9 technique (see below), in an enriched C57BL/6 background. Screening for the MarvelD3 deletion was realized by classical PCR with the GoTaq R2 Hot Start Green Master Mix (Promega) with fw (5′-TGATTGGTCTGGCGTCCTAAT-3′) and rv (5′-CCCCACCTACCTAGCCTTCT-3′), and with fw′ (5′-ACGGTGACGTGAGAGGAAAC-3′) and rv primers. MD3^+/+^, MD3^+/−^ and MD3^−/−^ females were mated and the day of vaginal plug was considered as embryonic day (E)0.5. Pregnant females were killed by cervical dislocation at the desired time point and embryos were collected. Adult MD3^+/+^, MD3^+/−^ and MD3^−/−^ mice were killed by cervical dislocation at 9 weeks of age for tissue collection. All mice were raised and treated according to the NIH Guide for Care and Use of Laboratory Animals. Experiments were approved by the University Animal Ethical Committee, UCLouvain (2012/UCL/MD/025, 2016/UCL/ MD/005 and 2020/UCL/MD/011), and followed the recommendations of the ARRIVE guidelines.

### CRISPR-Cas9 gene editing

The MarvelD3 knockout mouse was generated at the UCLouvain Transgenesis platform, by direct pronuclear microinjection of the Cas9 endonuclease (Integrated DNA Technologies), two MarvelD3-targetting crRNAs and a tracrRNA (0.61 pmol/μl crRNA, 0.61 pmol/μl tracrRNA, 30 ng/μl CAS9 in IDTE Buffer), using a FemtoJet 4i injector (Eppendorf). The Cas9 endonuclease binds to the tracrRNA, which is, in turn, recruited by the crRNA to a specific DNA sequence. Sequences targeted by the crRNAs were chosen using the CRISPR design tool (http://crispr.dbcls.jp/). crRNA1 (5′- TGT CGC CCT CGG ATC GTT TC-3′) targets a sequence in the first exon and crRNA2 (5′- GGA CCA GGG ACC GCC GAG GG -3′) localizes in the first intron of the gene. Injected C57BL/6 or B6D2 zygotes were incubated overnight at 37 °C, and 2-cell stage embryos were implanted into pseudopregnant CD1 females. Pups were screened for knockout alleles by genotyping PCR (Fig. 2b). One heterozygous male (C57BL/6) and two heterozygous females (B6D2) were selected based on Sanger sequencing of the PCR products to initiate the MarvelD3 knockout colony. Although crRNA1 initially targets a sequence within the first exon, genomic sequence was deleted until upstream of the transcription initiation site (Fig. 2a, scissors). Extended deletion was even better, as it should prevent transcriptional adaptation in response to non-sense mediated decay. The localization of crRNA2 was chosen within the first intron, as it is common to both MarvelD3 isoforms, and as it allows generation of a large, easily detectable deletion. The MarvelD3 knockout colony was further backcrossed into the C57BL/6 background.

### Immunofluorescence

Organs collected for immunolabeling were either fixed overnight (ON) in 4% paraformaldehyde at 4 °C and embedded in paraffin using a Tissue-Tek VIP-6 (Sakura), or fixed for 15 min, embedded in Tissue-TEK OCT (Sakura) and frozen in cold isopenthane. Sections of 7 µm were realized with a microtome (HM355S, Thermo Scientific) or cryostat (Cryostar NX70, Thermo Scientific), and deposited onto SuperFrost Plus slides. Slides with paraffin sections were deparrafinized, rehydrated, and unmasked with citrate buffer (10 mM, pH6) either rapidly heated for 10 min in a microwave (750Watts), or progressively heated in the PreTreatment Module (Lab Vision) for 2 h. Slides with OCT section were only unmasked. Preliminary steps further included tissue permeabilization (PBS/0.3% Triton X-100 for 5 min) and blocking (PBS/0.3% Triton X-100/10% Bovine Serum Albumin (BSA)/3% milk for 45 min). Primary antibodies listed in Table 1 were diluted in blocking solution and incubated either for 2 h at 37 °C, or ON at 4 °C. Secondary antibodies coupled to Alexa-488, -568 or -647 (Invitrogen) and fluorescent nuclear dye (Hoechst 33258; Sigma) were diluted in blocking solution without milk and incubated for 1 h at room temperature. Slides were finally mounted with Dako Mounting Medium, and scanned with the Pannoramic P250 Digital Slide Scanner, or observed with the Zeiss Cell Observer Spinning Disk (COSD) confocal microscope. For quantification of amylase- and insulin-positive epithelial cells, we used the image analysis platform HALO (Indica Labs) with its appropriate in-built algorithms. Three embryos from different litters were analysed.

**Table 1:** Immunofluorescence antibodies.

Antibody	Catalog #	Compagny	Isotype	Dilution	Other
Amylase	A8273	Sigma	Rabbit	1/300	
E-cadherin	610,182	BD Biosciences	Mouse IgG2a	1/300	
Endomucin	sc-8002	Santa Cruz	Rat	1/1000	
ERG	ab92513	Abcam	Rabbit	1/1000	
Ezrin	MS-661-P1	Themo Scientific	Mouse IgG1	1/300	
Insulin	A0564	Dako	Guinea pig	1/100	
Occludin	33–1500	Invitrogen	Mouse IgG1	1/200	15′ fixation, OCT embed
Pan laminin	L9393	Sigma	Rabbit	1/200	
PECAM	DIA310	Dianova	Rat	1/20	
Tricellulin	48–8400	Invitrogen	Rabbit	1/100	15′ fixation, OCT embed

### Glucose- and insulin-tolerance test

Two and four months-old MD3^+/−^ and MD3^−/−^ males and females were subjected to a glucose (GTT) and an insulin tolerance test (ITT), realized one week apart. For the GTT, mice were fasted for 16 h, with free access to water. Fasting glycaemia was measured at time 0′ with a glucometer (Contour XT, Bayer) on a droplet of blood obtained by tail vain incision. Glucose (Merck) was diluted in sterile PBS and a single dose of 2 mg/g of body weight was injected intraperitoneally. Blood glucose levels were assessed after 15′, 30′, 60′, 90′ and 120′. Mice were not fasted prior to ITT. Insulin (Roche) was diluted in sterile PBS and injected intraperitoneally at a dose of 0.5 IU/g of body weight. Glycaemia was measured before insulin injection (0′), and after 15′, 30′ and 60′. Hypoglycaemia was then reverted by injection of a single shot of the glucose solution. Data have been generated from 9 individuals.

### RNAScope in situ hybridization assay

The RNAScope technique makes use of a Z-shaped probe to detect a specific mRNA on tissue sections. The lower part of the “Z” recognizes the target sequence, while the upper part of the “Z” recruits a cascade of signal amplification molecules, ending with the binding of an alkaline phosphatase-labelled probe. Incubation with its substrate (Fast Red), leads to the formation of a red precipitate that can be visualized using a bright field or fluorescence microscope.

Tissue samples harvested for RNAScope in situ hybridization (ISH) were fixed overnight (ON) in 4% paraformaldehyde and embedded in paraffin using a Tissue-Tek VIP-6 (Sakura). Sections of 5 µm, obtained with a microtome (HM355S, Thermo Scientific) were deposited onto SuperFrost Plus slides and air-dried ON. *MarvelD3* mRNA expression was evaluated using the RNAScope 2.5 High Definition RED Assay kit (322350, Advanced Cell Diagnostics). Briefly, slides were baked for 1 h at 60 °C in the HybEZ II oven (Advanced Cell Diagnostics), deparaffinized and dehydrated in clean solutions. Inhibition of endogenous peroxidases, target retrieval and protease treatment were then performed. Probes for *MarvelD3* and *Ubc* (positive control) were hybridized on adjacent sections, for 2 h at 40 °C in the HybEZ II oven. Signal amplification was obtained with 6 amplification steps according to the manufacturer’s protocol (duration of treatment and temperature). mRNAs were revealed using alkaline phosphatase Fast Red chromogenic staining. Sections were counterstained with Mayer’s haematoxylin solution, and scanned using the Pannoramic P250 Slide Scanner. Three embryos from the same litter were analysed.

### RT-qPCR and statistical analysis

Tissues collected from embryos until day E15.5 were collected and immediately snap-frozen in liquid nitrogen. Total RNA was extracted using TRIzol Reagent (Thermo Scientific) followed by phenol/chloroform extraction, as described^27^. E17.5 pancreata and 2 × 2 mm pieces of adult pancreata were submerged into RNAlater Stabilization Solution (Thermo Scientific) and frozen in liquid nitrogen. Total RNA was extracted using the REliaPrep RNA Tissue Miniprep System (Z6111, Promega), according to the manufacturer’s instructions for small (≤ 5 mg) fibrous tissue samples. Reverse transcription was realized on 500 ng of total RNA with random hexamer primers and the M-MLV Reverse Transcriptase (Invitrogen). The Kapa SYBR Fast qPCR kit (Sopachem) was used for RT-qPCR on the obtained cDNAs. Primers sequences for the different genes are listed in Table 2. Data were analysed according to the Livak method (ΔΔCT) and were represented as mean ± standard deviation of mRNA Log_2_ fold changes relative to the expression of housekeeping gene *β-actin*, or of *Rpl27* (Fig. 1A). In dot plots, each symbol represents one mouse of a particular MarvelD3 genotype: green circles for wild-type, orange squares for heterozygous, and red triangles for knockout. Between 4 and 7 samples were analysed. Statistical analyses were realized using non-parametric tests: Mann–Whitney for comparison of 2 conditions, Kruskall-Wallis for more conditions. In case of a significant (i.e. *p* < 0.05) Kruskall-Wallis test, Mann–Whitney tests were realized between control and tested conditions to acknowledge a potential significant difference, which is represented by * when *p* < 0.05 and ** when *p* < 0.01.

**Table 2:** RT-qPCR primers.

Primer name	Forward sequence	Reverse sequence
*Actb*	5′-TCCTGAGCGCAAGTACTCTGT-3′	5′-CTGATCCACATCTGCTGGAAG-3′
*Amy*	5′-GTGGTCAATGGTCAGCCTTT-3′	5′-TTGCCATCGACCTTATCTCC-3′
*Atp1a*	5′-TTCACCTGCCATACAGCGTT-3′	5′-GCAGTGTGTGGCACAATGTT-3′
*Cdh1*	5′-AGGGAGCTGTCTACCAAAGTG-3′	5′-GGAAACATGAGCAGCTCTGGG-3′
*Cdh5*	5′-GGATGTGGTGCCAGTAAACC-3′	5′-ACCCCGTTGTCTGAGATGAG-3′
*Cldn1*	5′-CCACCATTGGCATGAAGTGC-3′	5′-AGAGGTTGTTTTCCGGGGAC-3′
*Cldn3*	5′-ACTGCGTACAAGACGAGACG-3′	5′-GTAGTCCTTGCGGTCGTAGG-3′
*Cldn4*	5′-CCATGGAACCCTTCCGTTGA-3′	5′-GCAAGACAGTGCGGAAAAGG-3′
*Cldn7*	5′-CATGTACAAGGGGCTCTGGA-3′	5′-TGGACAGGAGCAAGAGAGCA-3′
*Cpa*	5′-CTCCTGACAAGGAGGAGCTG-3′	5′-ATAGTGCTCCCACTGGCTTG-3′
*Flk1*	5′-GCATGGAAGAGGATTCTGGA-3′	5′-CGGCTCTTTCGCTTACTGTT-3′
*Gcg*	5′-GCACATTCACCAGCGACTACA-3′	5′-CGGTTCCTCTTGGTGTTCATC-3′
*Ins2*	5′-CAGGTGACCTTCAGACCTT-3′	5′-GGGTCTAGTTGCAGTAGTTC-3′
*MD3.1*	5′-AACGGGCTTCGGAAGATAC-3′	5′-CCCCGTGGAATTGTAAGAGA-3′
*MD3.2*	5′-AACGGGCTTCGGAAGATAC-3′	5′-TGAGAACCATGGCATTCAAA-3′
*Ocln*	5′-TGATGCAGGTCTGCAGGAGT-3′	5′-CACCATCCTCTTGATGTGCG-3′
*Pdx1*	5′-AAGAGCCCCAACCGCGTCCAGC-3′	5′-AGTACGGGTCCTCTTGTTTTC-3′
*Pecam1*	5′-ATAGGCATCAGCTGCCAGTC-3′	5′-TCCGCTCTGCACTGGTATTC-3′
*Pnlip*	5′-CATACCCCTGCGCTTCCTAC-3′	5′-CTTCTGTGGCTCCACACTTG-3′
*Prox1*	5′-CCGACATCTCACCTTATTCAG-3′	5′-TGCGAGGTAATGCATCTGTTG-3′
*Prss1*	5′-TTCTGATCCTAGCCCTTGTG-3′	5′-TGATAGGGGACAGAACTCTC-3′
*Ptf1a*	5′-TGCCATCGAGGCACCCGTTC-3′	5′-TGAGCTGTTTTTCATCAGTCCAG-3′
*Rbpj*	5′-GGTCCCAGACATTTCTGCAT-3′	5′-GGAGTTGGCTCTGAGAATCG-3′
*Rbpjl*	5′-CAGAGCATGCCATCATCCTA-3′	5′-AGTCCCATGTAACCGCAGAC-3′
*Rpl27*	5′-GCCCTGGTGGCTGGAATTGACC-3′	5′-AAACTTGACCTTGGCCTCCCGC-3′
*Sox9*	5′-CAAGACTCTGGGCAAGCTCTG-3′	5′-TCCGCTTGTCCGTTCTTCAC-3′
*Tric*	5′-TGCCTGACTACGTGGCAAAA-3′	5′-ACCAAGCGGAAGATGACCTG-3′

### Western blotting

Pancreas and kidney tissue samples were homogenized in classical RIPA Buffer (Fig. 3D) or Urea Lysis Buffer (Urea 6 M, NaCl 100 mM, Tris 50 mM pH8, 1% Triton X-100, 0,1% SDS) (Fig. 5A et S3A) using an Ultra-Turrax T10 (Ika). Total protein concentration was assessed via a bicinchoninic acid assay. 50 μg of proteins were diluted 3 × into urea Sample Buffer (Urea 6 M, Tris–HCl 0.188 M pH6.8, DTT 150 mM, 6% SDS, 30% glycerol, 0.003% bromophenol blue), and deposited onto a 8% SDS polyacrylamide gel (3% stacking gel). Proteins were separated by SDS-PAGE and transferred onto a PVDF membrane. The membrane was blocked in PBS containing 5% milk and 0.1% Tween-20 for 45 min, and incubated overnight at 4 °C with primary antibodies against β-Actin (1/200, A2066, Sigma), Tubulin (1/500, T6199, Sigma), JNK (1/1.000, 9258, Cell Signalling), phospho-JNK (1/1.000, 4668, Cell Signalling), c-Jun (1/1.1000, 9165, Cell Signalling), phospho Ser63-c-Jun (1/1.000, 2361, Cell Signalling), phosphor Ser73-c-Jun (1/1.000, 3270, Cell Signalling) or MarvelD3 (1/5.000). The rabbit polyclonal antibody against mouse MarvelD3 was raised against the R T R P R E R D P D R R P H P D R D H C peptide. The antibody was affinity purified using the same peptide immobilized on epoxy-activated Sepharose. Afterwards, the membrane was washed with PBS/0.1% Tween-20 and the appropriate secondary antibody coupled to horseradish peroxidase was added for 1 h (1/10.000 in PBS/T with 0.5% milk). Immunoreactive proteins were visualized by chemiluminescence (Supersignal West Femto substrate, Thermo Scientific), and photographed using the Fusion Solo S (Vilber Lourmat). All samples analysed were shown in the blots. Bands of interest were quantified using ImageJ Software and normalized on tubulin signals.

## Supplementary information

Supplementary Information.

## Data Availability

Materials, data and associated protocols are available.
