# Supplementary material for: Spatio-temporal expression pattern and role of the tight junction protein MarvelD3 in pancreas development and function
Source: Sci Rep. 2021 Jul 15;11:14519. doi: 10.1038/s41598-021-93654-2 (PMC8282860; doi:10.1038/s41598-021-93654-2)
Supplement: Supplementary file 1 — Supplementary Information. [file 41598_2021_93654_MOESM1_ESM.pdf]

Figure Sup. 1

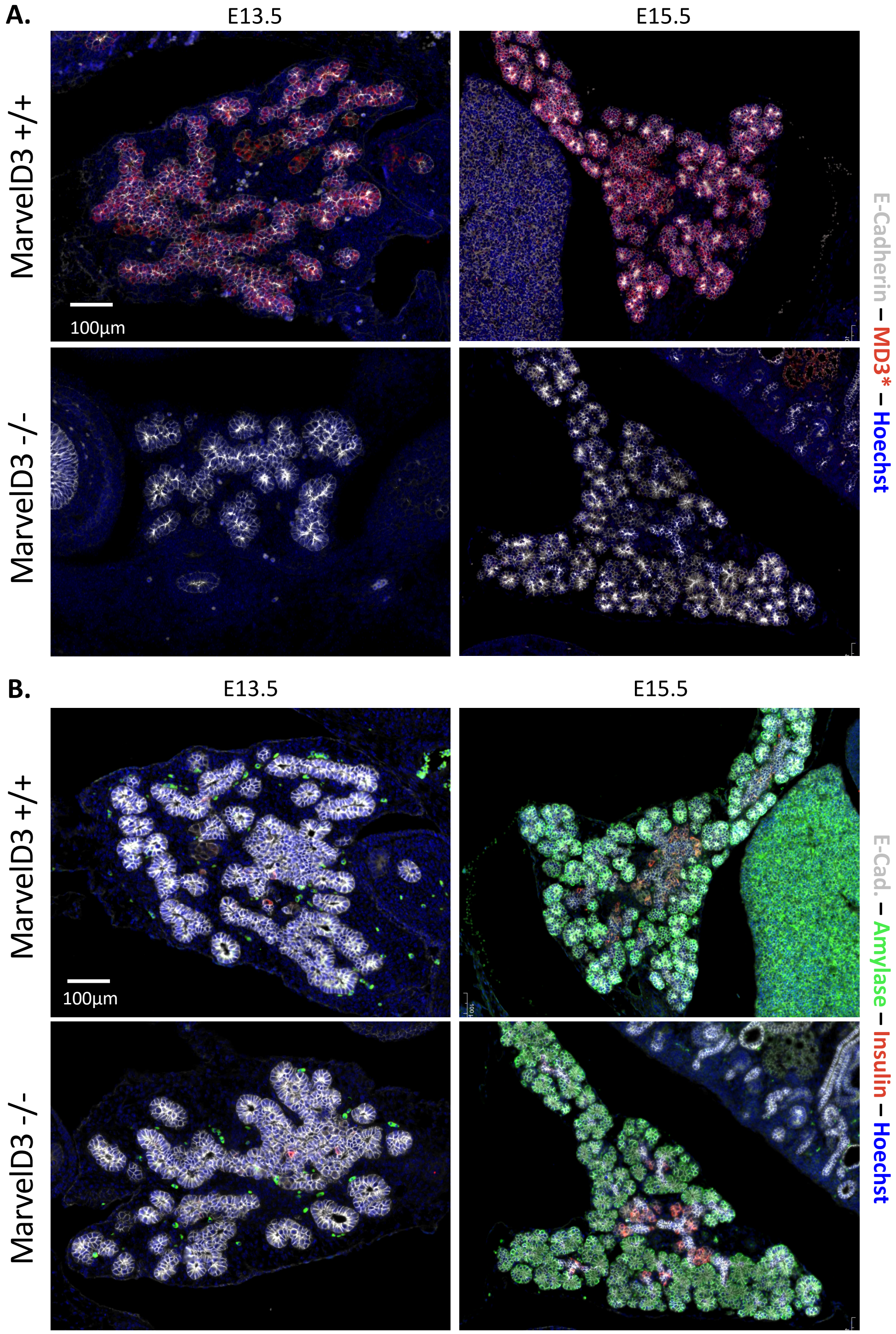

Figure S1: Evaluation of pancreas differentiation in wild-type and MarveldD3-knockout tissues during embryonic development.

A) Fluorescent RNAScope® Assay to detect MarvelD3 mRNA (MD3*, red) in the pancreatic epithelium (E-Cadherin^{+}, red) of wild-type (+/+) and knockout (-/-) embryos (E13.5-E15.5). MarvelD3 is expressed in all epithelial (i.e. acinar, ductal and endothelial) cells of the +/+ pancreas at E13.5. MarvelD3 probe does not hybridize in the knockout tissue. **B)** Immunolabeling of acinar (E-Cadherin^{+}, white and amylase^{+}, green), ductal (E-Cadherin^{+}, white) and endocrine (E-Cadherin^{+}, white and insulin^{+}, red) cells in the developing pancreas (E13.5-E15.5) of wild-type (+/+) and knockout (-/-) embryos. Acinar differentiation, assessed by the number of amylase-expressing cells, is not modified in E13.5 embryos lacking MarvelD3. Acinar and endocrine differentiation is similar in +/+ and -/- tissues at E15.5.

Figure Sup. 2

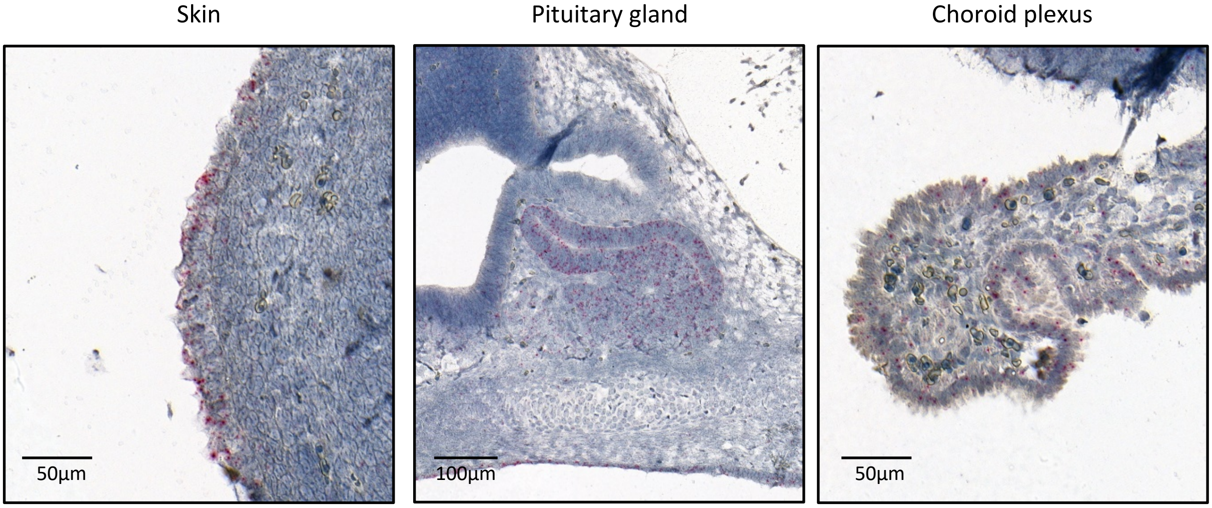

Figure S2: MarvelD3 is expressed in ectoderm-derived tissues of the mouse embryo.
RNAScope® *in situ* hybridization in MarvelD3 E13.5 wild-type tissues of ectodermal origin. The MarvelD3 probe hybridizes in cells of the epidermis, the pituitary gland and the ependymal epithelium of the choroid plexus.

Figure Sup. 3

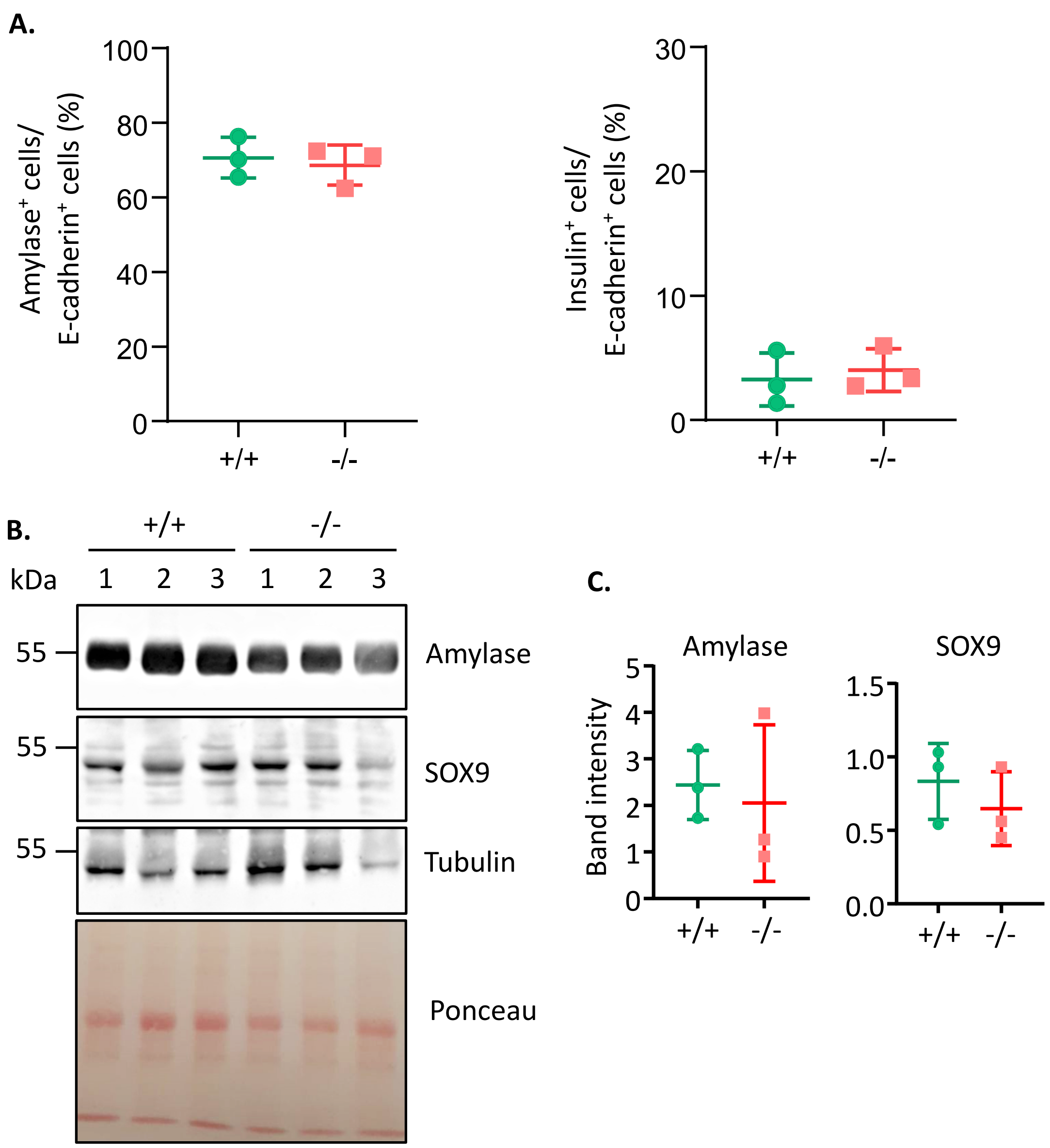

Figure S3: MarvelD3 absence does not impact on pancreas differentiation.

A) Morphometric analyses of E15.5 pancreatic sections stained for amylase, insulin and E-cadherin shown in Figure 4C (n=3). HALO software was used to segment nuclei and quantify the number of cells expressing amylase, insulin and E-cadherin. Results are presented as the percentage of epithelial cells expressing amylase (at left) and insulin (at right). B) Western blotting of Amylase and of Sox9 in protein extracts from 3 adult wild-type (+/+) and 3 MarvelD3 knockout (-/-) pancreata. Tubulin and Ponceau staining are showed as loading control. Full-length blots/gels are presented in Supplementary Figure 7. C) No significant differences in the abundance of these proteins was observed. Quantification was done on Tubulin, and Ponceau is shown as another loading control.

Figure Sup. 4

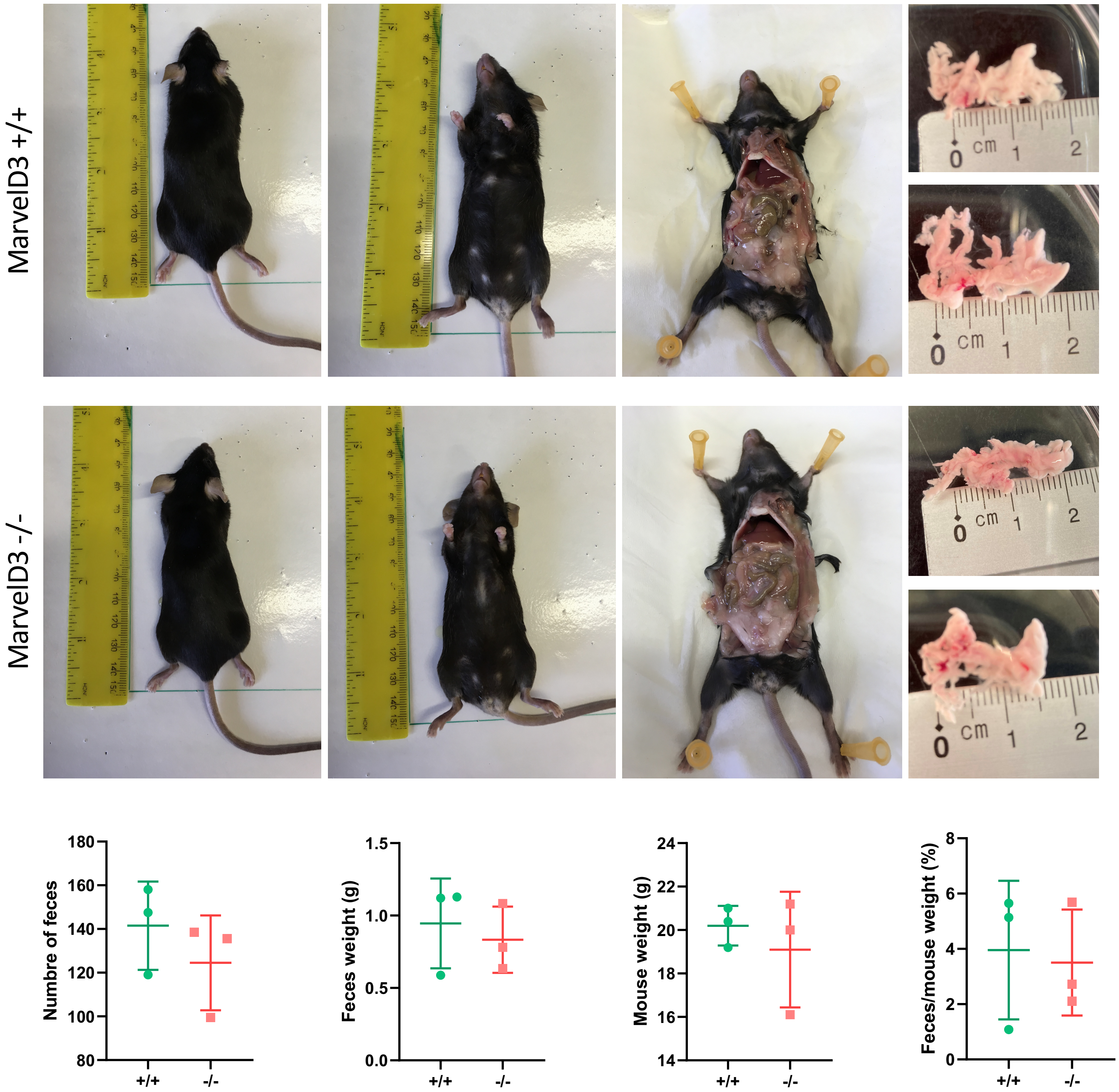

Figure S4: MarvelD3 knockout are undistinguishable from control mice.
Pictures of control and MarvelD3 knockout mice and pancreas (n=2). Number and weight of faeces produced over a period of 24h (mean of 2 collections), weight of mice and ratio of faeces/mice (n=3).

Figure Sup. 5

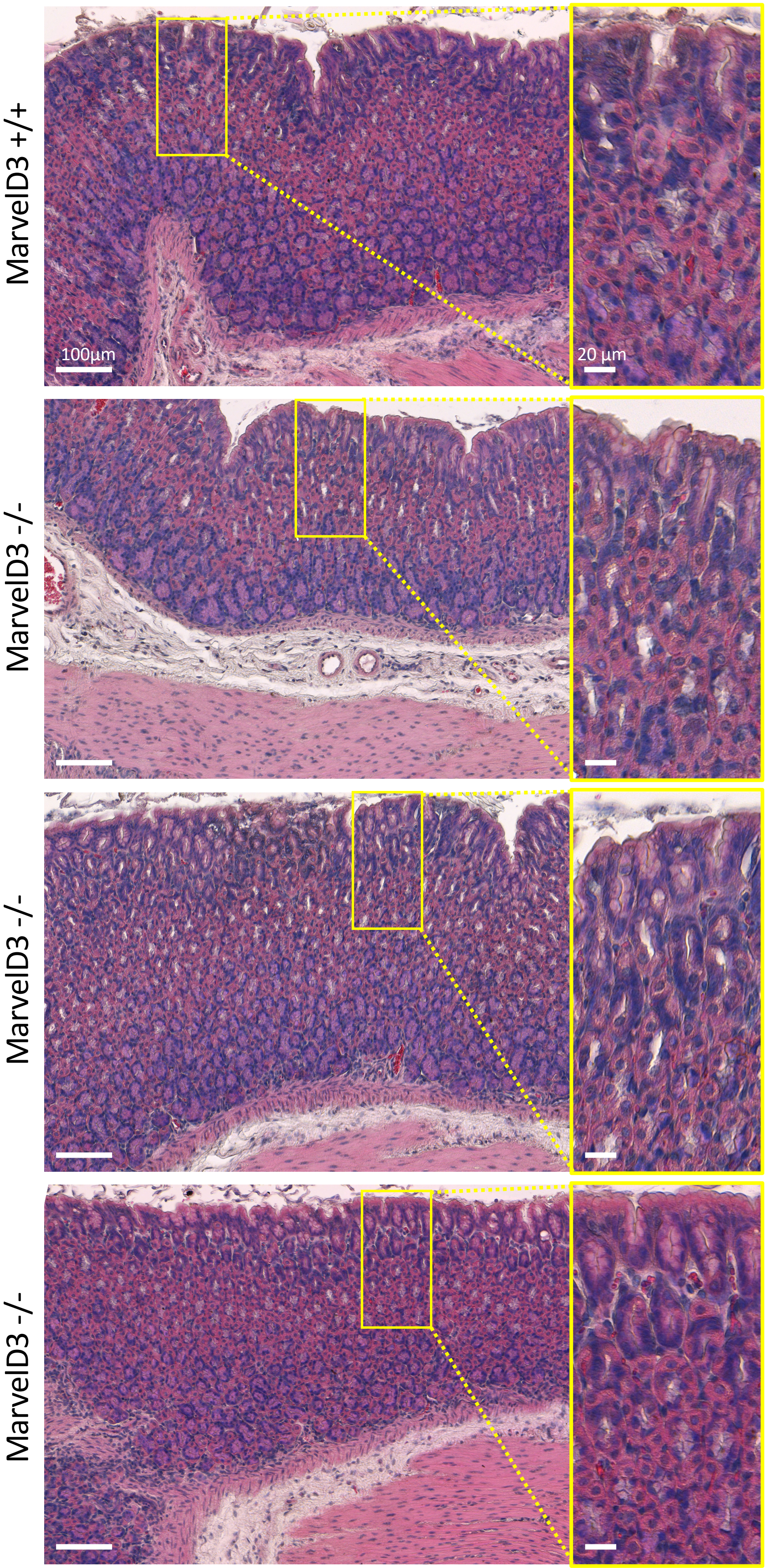

Figure S5: MarvelD3 absence does not impact on paracellular permeability.
H&E stained sections of 1-year old stomach from one control and three knockouts at low and high magnification (at right) reveal no immune infiltrate in the stomach mucosae.

Figure Sup. 6

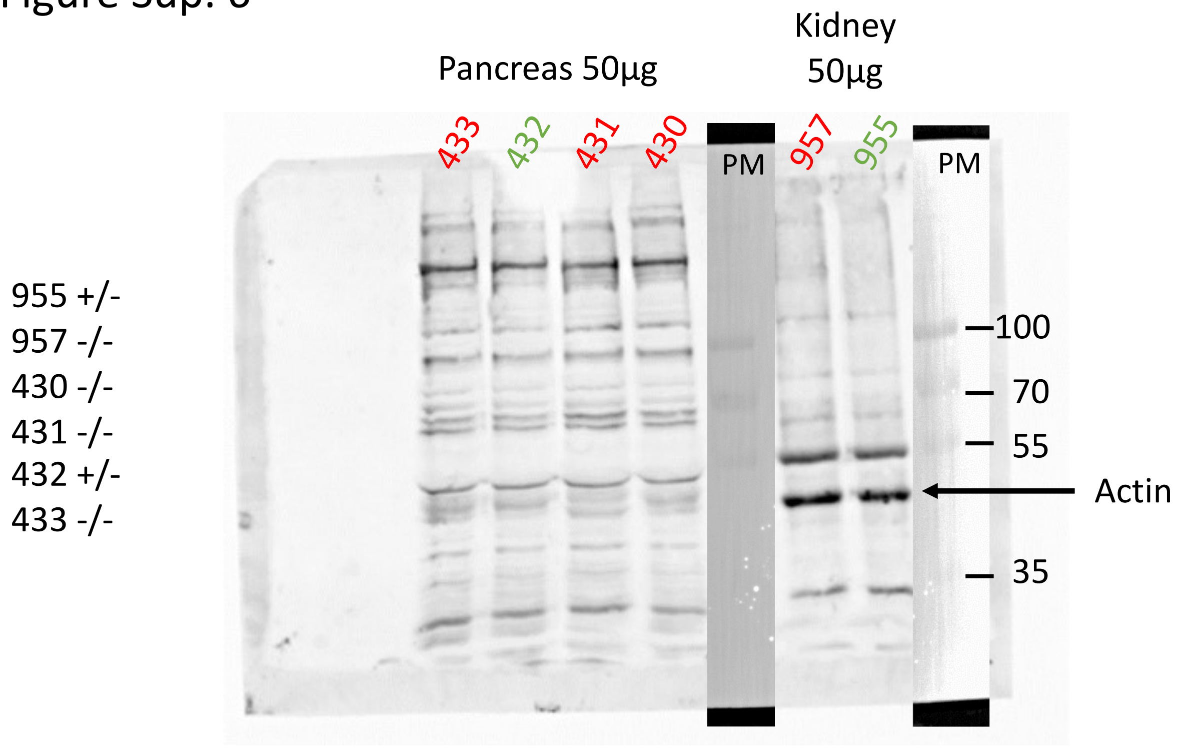

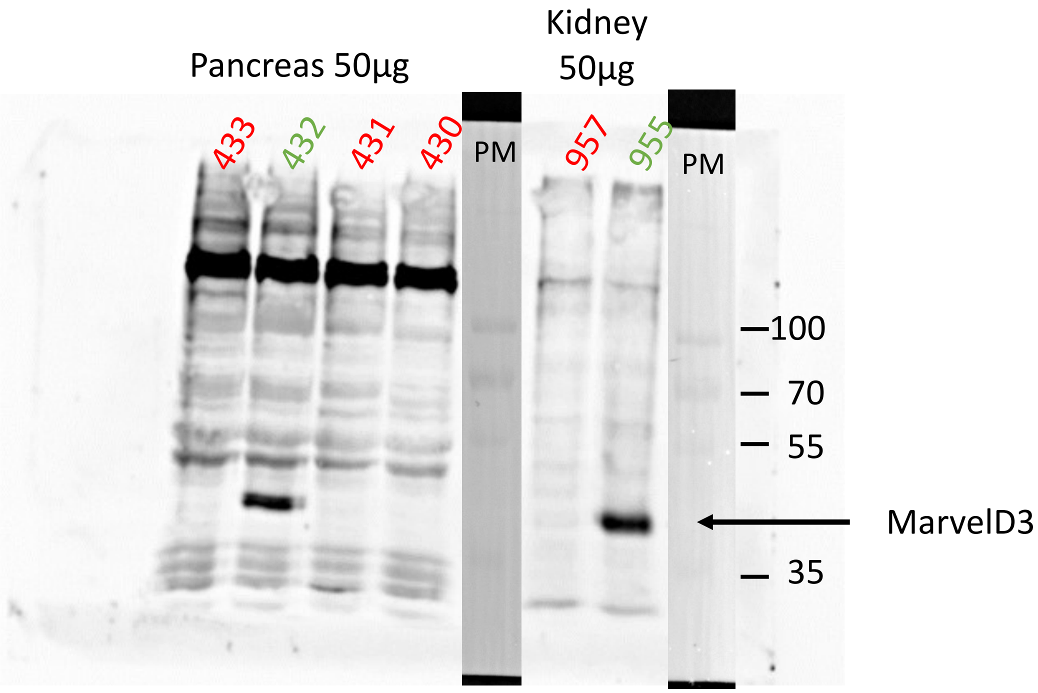

Figure S6: full-length blots/gels for MarvelD3 and Actin.

Chemiluminescent signal of the full blots on pancreatic and kidney extracts. PM, lane with the coloured molecular weight markers. In figure 3D, samples 433 and 432 (pancreas) and samples 955 and 957 (kidney) were selected, cropped and flipped vertically.

Figure Sup. 7

Amylase

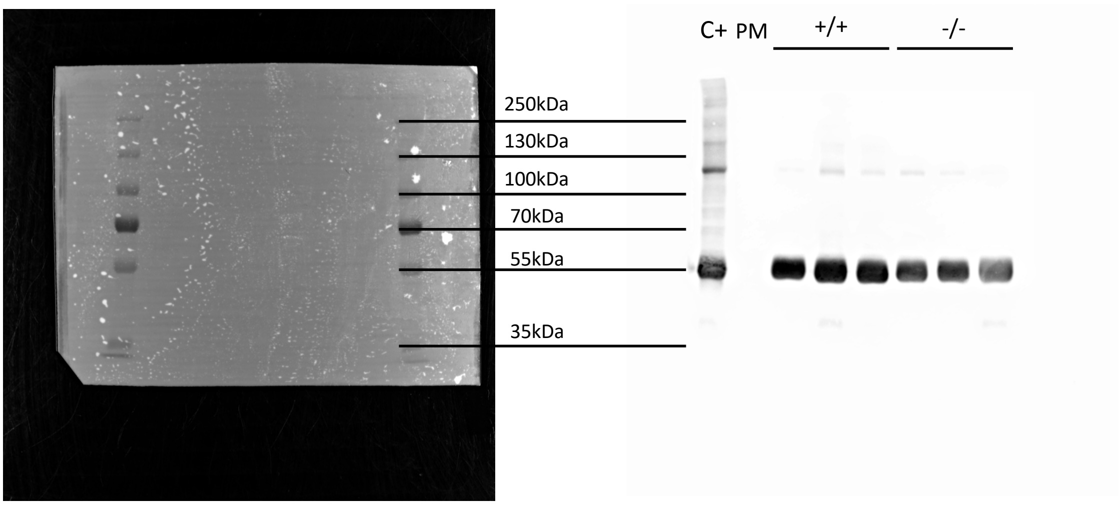

SOX9

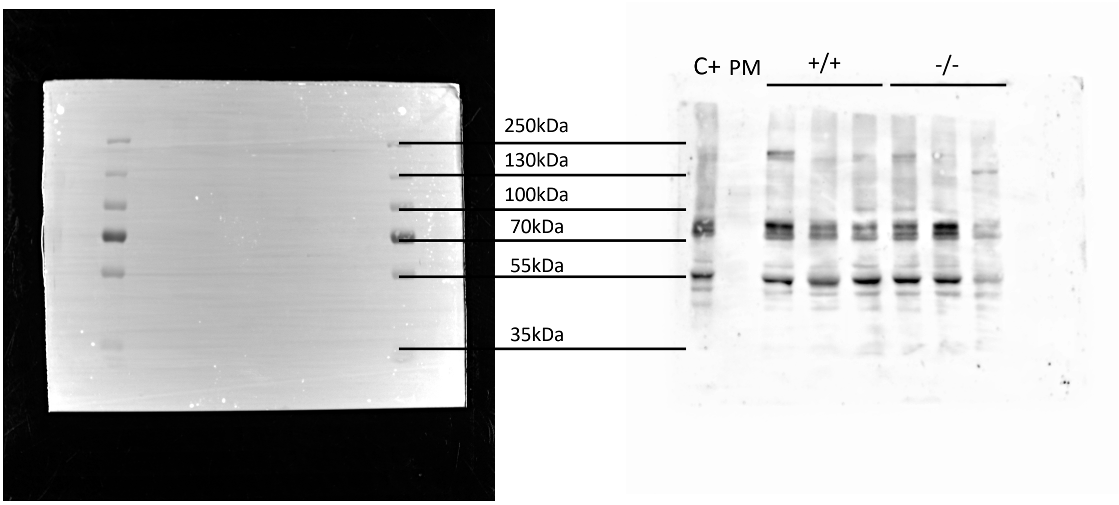

Tubulin

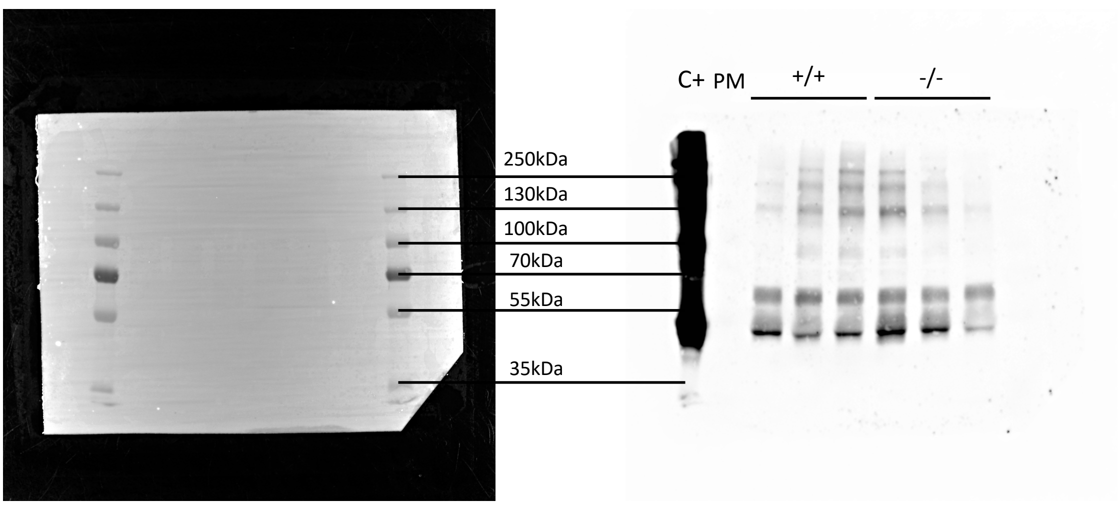

Figure S7: full-length blots/gels for amylase, Sox9 and Tubulin.
Membrane with coloured molecular weight markers are shown at left. Chemiluminescent signal are shown at right. C+, positive control used in a preliminary experiment. PM, lane with the coloured molecular weight markers.

Figure Sup. 8

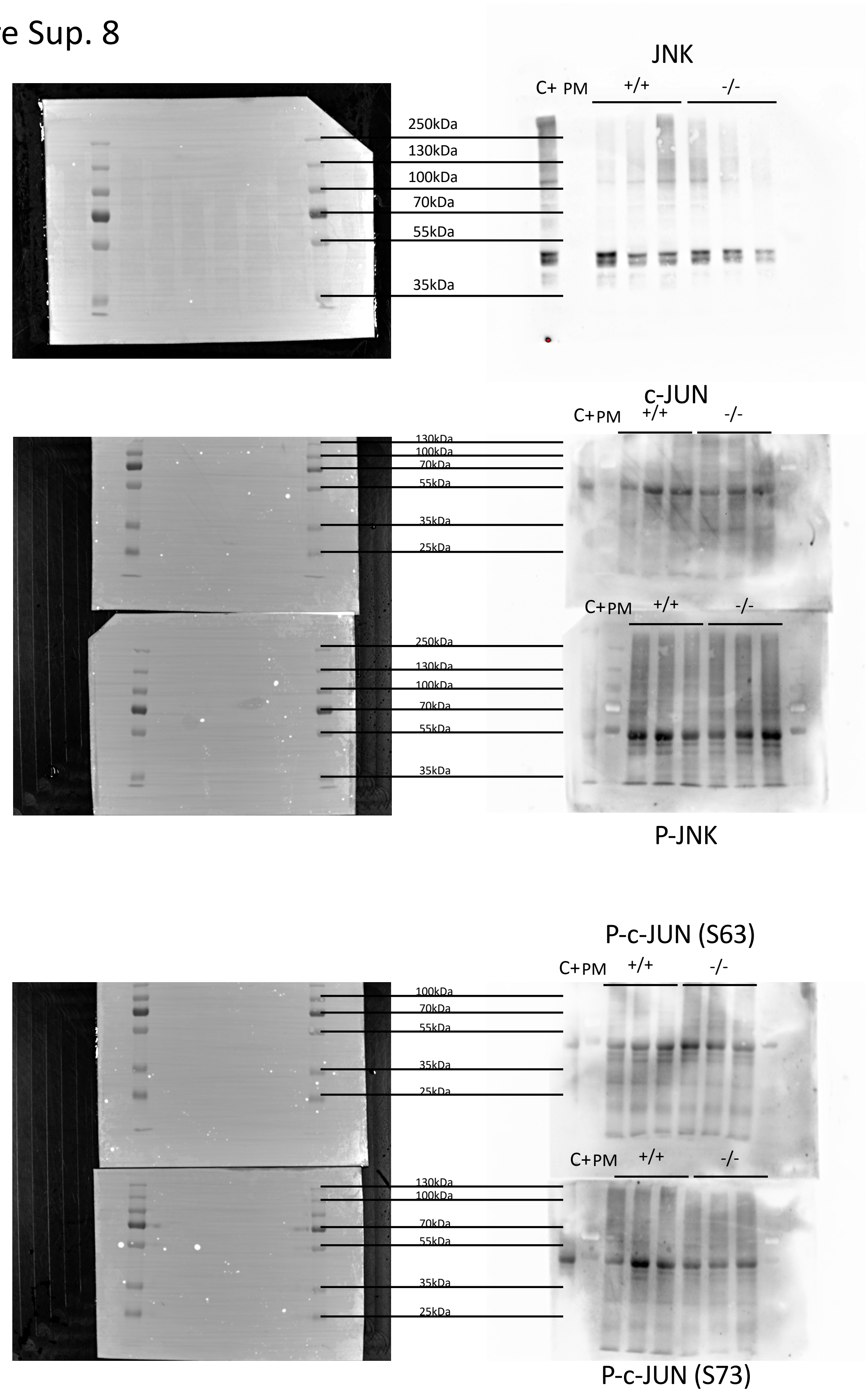

Figure S8: full-length blots/gels for JNK, c-Jun, P-JNK, P-c-Jun (S63) and P-c-Jun (P73). Membrane with coloured molecular weight markers are shown at left. Chemiluminescent signal are shown at right. C+, positive control used in a preliminary experiment. PM, lane with the coloured molecular weight markers.
